# The Impact of Oxygen on Metabolic Evolution: A Chemoinformatic Investigation

**DOI:** 10.1371/journal.pcbi.1002426

**Published:** 2012-03-15

**Authors:** Ying-Ying Jiang, De-Xin Kong, Tao Qin, Xiao Li, Gustavo Caetano-Anollés, Hong-Yu Zhang

**Affiliations:** 1 National Key Laboratory of Crop Genetic Improvement, College of Life Science and Technology, Huazhong Agricultural University, Wuhan, China; 2 Center for Bioinformatics, Huazhong Agricultural University, Wuhan, China; 3 State Key Laboratory of Agricultural Microbiology, Huazhong Agricultural University, Wuhan, China; 4 School of Life Sciences, Shandong University of Technology, Zibo, China; 5 Evolutionary Bioinformatics Laboratory, Department of Crop Sciences, University of Illinois, Urbana, Illinois, United States of America; JCVI, United States of America

## Abstract

The appearance of planetary oxygen likely transformed the chemical and biochemical makeup of life and probably triggered episodes of organismal diversification. Here we use chemoinformatic methods to explore the impact of the rise of oxygen on metabolic evolution. We undertake a comprehensive comparative analysis of structures, chemical properties and chemical reactions of anaerobic and aerobic metabolites. The results indicate that aerobic metabolism has expanded the structural and chemical space of metabolites considerably, including the appearance of 130 novel molecular scaffolds. The molecular functions of these metabolites are mainly associated with derived aspects of cellular life, such as signal transfer, defense against biotic factors, and protection of organisms from oxidation. Moreover, aerobic metabolites are more hydrophobic and rigid than anaerobic compounds, suggesting they are better fit to modulate membrane functions and to serve as transmembrane signaling factors. Since higher organisms depend largely on sophisticated membrane-enabled functions and intercellular signaling systems, the metabolic developments brought about by oxygen benefit the diversity of cellular makeup and the complexity of cellular organization as well. These findings enhance our understanding of the molecular link between oxygen and evolution. They also show the significance of chemoinformatics in addressing basic biological questions.

## Author Summary

Elucidating the link between the rise of planetary oxygen and biological evolution is a challenging topic in evolutionary biology. Previous studies in this area were dominated by biological investigations. The recent simulations of metabolic networks under anaerobic or aerobic conditions revealed that aerobic metabolism gave rise to 1,000+ new reactions. Since metabolites are small molecules and metabolic reactions are basically chemical reactions, we think that the impact of oxygen on metabolic evolution can be well studied by chemoinformatics. In this paper, we use chemoinformatic methods to perform a comprehensive comparative analysis of the chemical structures, properties and reactions of anaerobic and aerobic metabolites. It was found that aerobic metabolism has considerably expanded the structural space of metabolites by inventing 130 novel molecular scaffolds. Moreover, aerobic metabolism also helped organisms to explore a new chemical space by increasing the hydrophobicity and rigidity of metabolites. Since hydrophobic metabolites are fitting to modulate membrane functions and to serve as transmembrane signaling factors, these metabolic innovations definitely benefit the establishment of complex cellular organizations. The present findings not only help to understand the molecular link between oxygen and evolution but also suggest that chemoinformatics is of special value in addressing some basic biological questions.

## Introduction

Accumulating biological and geological evidence indicates that oxygen has had a great impact on biological evolution [1]–[3]. The atmosphere and seas of our planet were anaerobic ∼3 billion years ago [4]–[6], at a time when life was not yet diversified [7]. However, the rise of atmospheric oxygen triggered at least the appearance of eukaryotes [7]. Later on in evolution, molecular oxygen continued to play a critical role. For instance, the substantial rise in atmospheric oxygen 750 million years ago was probably responsible for the Cambrian explosion of animal diversity [8], [9]. The rise of atmospheric oxygen during the Devonian also coincided with radiations of terrestrial plants and large predatory fish [10]. Finally, the elevation of oxygen levels over the past 205 million years could have increased animal body size [11].

Elucidating the molecular link between the rise of oxygen and biological evolution has been one of the most challenging topics in evolutionary biology. A straightforward explanation to this link is that aerobic respiration is much more efficient than anaerobic respiration in generating ATP [12]. Recent analyses about the evolution of protein structures revealed that oxygen has largely expanded protein structural space [2], [3]. This implies an accompanied expansion of their chemical and functional space. Indeed, 100,000 simulations of metabolic networks under anaerobic or aerobic conditions revealed that molecular oxygen enabled over 1,000 more metabolic reactions than reactions in its absence [13]. While a small part of these aerobic reactions have anaerobic counterparts, most of them are new and give birth to new sets of metabolites. All oxygen-consuming reactions are irreversible [14]. Consequently, the aerobic reactions are thermodynamically more efficient [15]. However, the impact of oxygen on metabolic evolution goes beyond thermodynamic considerations. In particular, the new aerobic metabolites may exhibit some functional innovations, which may provide important clues to understanding the influence of global oxygenation on biological evolution.

Since metabolites are small molecules and metabolic reactions are basically chemical reactions, they could be best dissected by chemoinformatics [16]–[18], a discipline dedicated to the storage, management, analysis, dissemination and usage of chemical information. Here we use chemoinformatic methods to perform a comprehensive comparative analysis of the chemical structures, properties and reactions of anaerobic and aerobic metabolites. Our results reveal how oxygen had an impact on metabolic evolution in chemical space and provide new insights into the relationships between the rise of oxygen and biological evolution.

## Results/Discussion

### Profiles of anaerobic and aerobic metabolic networks

The aerobic and anaerobic metabolic networks simulated by Raymond and Segrè consist of 1,145 anaerobic and 454 aerobic reactions, from which 1,326 anaerobic metabolites and 538 aerobic metabolites can be identified [13]. The origins of these metabolic reactions are diverse, with ∼50% of the aerobic reactions being specific to eukaryotes. Many of these are typical of animals and plants. While most of biological diversity is microbial and could by that token incorporate a sampling bias in the metabolites we examine, a study of functional annotations of protein structures in almost a thousand sequenced genomes show that patterns of enzymatic diversity are remarkably conserved throughout life [19]. Most of the proteomic repertoire was spent on metabolic processes, but with few exceptions, overall metabolic functions were highly conserved across all organisms of Archaea, Bacteria and Eukarya. Consequently, we do not expect that sampling bias that exists in the enzymatic toolkit will invalidate major conclusions of our study.

An analysis of metabolic network structure reveals the existence of some predominant modules in the aerobic and anaerobic pathways. For example, 48 and 19 major modules account for 80.2% and 80.8% of anaerobic metabolites and aerobic metabolites, respectively (Tables S1 and S2). Analysis of the metabolic reactions of the simulated networks reveals that the 81 initial reactants for the 48 major anaerobic modules participate on average in 8.1 reactions (Table S1). In comparison, the 23 initial reactants for the 19 major aerobic modules participate on average in only 3.4 reactions and these reactants are distant from the anaerobic central metabolites (with an average distance of 8.7 reactions) (Table S2). This suggests that reactions in anaerobic pathways tend to start from the center of metabolic networks. In contrast, oxygen-dependent pathways tend to start from the periphery of the anaerobic network, consistent with previous observations (Figure S1) [13].

**Figure 1:**
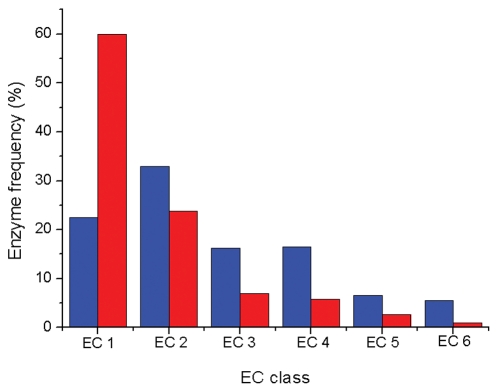
Frequency of anaerobic (in blue) and aerobic (in red) enzymes in six categories (EC1∼EC6).

At protein level, prior studies revealed that at least 31 folds are exclusively used by aerobic enzymes, most of which (90%) use oxygen explicitly [2]. A comparison of enzyme activities showed that oxidoreductases are predominant in aerobic but not in anaerobic enzymes (Figure 1). This is expected since oxygen prefers to participate in redox reactions. Since one noteworthy feature of oxidoreductases is their dependence on cofactors to perform the redox reactions, we analyzed cofactor usage for 154 anaerobic and 121 aerobic oxidoreductases (Table 1). In anaerobic enzymes, organic cofactors (*e.g.*, NAD(H), NADP(H) and FAD) were more prevalent than metallic counterparts (*e.g.*, iron, copper and molybdenum), while in aerobic enzymes, metallic redox cofactors were more popular. This mirrors the poor bioavailability of copper and molybdenum in the anaerobic world [20]–[22] and also agrees with prior conclusions that primitive redox enzymes (which are anaerobic enzymes) mainly used organic cofactors in catalysis [23]. Moreover, while NAD(H) is more common than NADP(H) in anaerobic enzymes, the opposite is true for aerobic enzymes (Table 1). This observation is particularly relevant since isocitrate dehydrogenases tend to generate NADP(H) rather than NAD(H) when adapting to environments with acetate [24]. Since acetate abundance was linked to the rise of oxygen [25], the prevalence of NADP(H) in aerobic enzymes is likely the result of oxygen-facilitated evolution.

**Table 1:** Cofactor usage in anaerobic and aerobic oxidoreductases.

Cofactors^a^	Cofactor occurrence (percentage)	
	Anaerobic enzymes	Aerobic enzymes
NAD(H)**	70 (45.5%)	12 (9.9%)
NADP(H)	46 (29.9%)	32 (26.4%)
NAD/P(H)	20 (13.0%)	15 (12.4%)
FAD	15 (9.7%)	22 (18.2%)
Ascorbate**	0 (0.0%)	8 (6.6%)
Fe-S	8 (5.2%)	9 (7.4%)
Iron**	4 (2.6%)	32 (26.4%)
Heme**	2 (1.3%)	32 (26.4%)
Molybdenum	1 (0.6%)	5 (4.1%)
Copper*	0 (0.0%)	6 (5.0%)

a derived from UniProt.

**: χ^2^ significance at *P*<0.01.

*: χ^2^ significance at *P*<0.05.

Thus, anaerobic and aerobic metabolic networks are remarkably distinct and must exhibit marked differences in their chemical space. This includes the structural, chemical and reaction properties of their metabolites.

### The impact of oxygen on metabolic evolution in structural space

**Figure 2:**
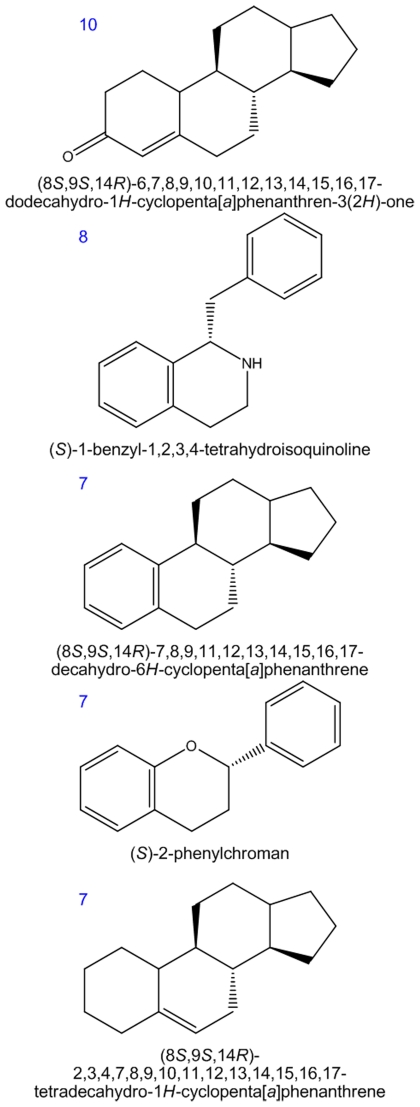
Most prevalent scaffolds of aerobic metabolism. The occurrence number of the scaffold in aerobic metabolite space is labeled at the top left of the structure.

In order to explore the impact of oxygen on metabolic evolution in structural space, we first calculated the scaffolds (chemical cores) for 1,174 anaerobic metabolites and 520 aerobic metabolites with clearly defined structures. Since different molecules can share the same scaffold, scaffolds are much more conserved than overall chemical structures [26]. Our calculation reveals that 204 scaffolds are used by the 1,174 anaerobic metabolites we studied. This represents an average of 0.174 scaffolds per anaerobic metabolite. In turn, 165 scaffolds are used by 520 aerobic metabolites, which represent an average of 0.317 scaffolds per aerobic metabolite. Since both types of metabolites share only 34 scaffolds, aerobic metabolism gives rise to a large number (>130) of novel scaffolds and these are more represented in its metabolic reactions. In particular, steroid, quinoline and flavonoid scaffolds are the most prevalent (Figure 2).

**Figure 3:**
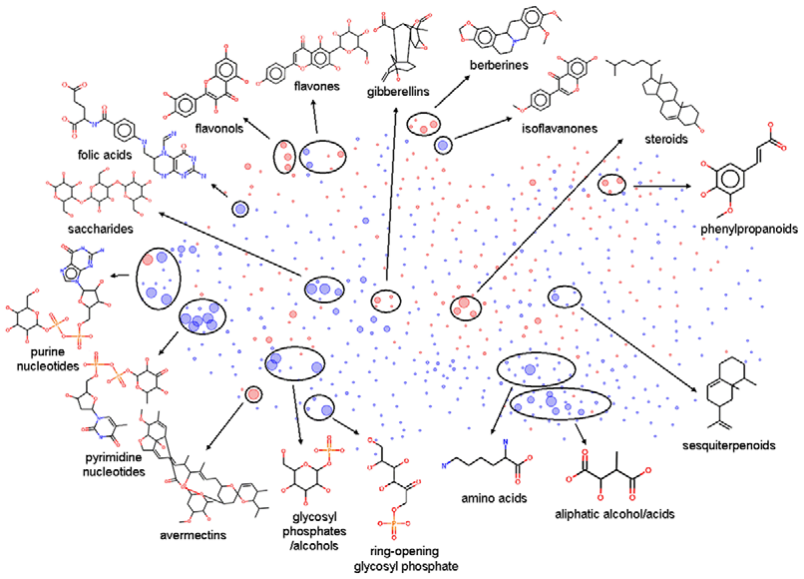
Structural cluster map of anaerobic and aerobic metabolites (represented with blue dots and red dots, respectively). In this map, the distance of two compounds is determined by their Tanimoto similarity (based on shared substructural fragments). Similar compounds are pooled together into clusters. The dot size indicates the number of the clustered compounds (with Tanimoto similarity >0.85). The largest dots represent clusters containing more than 10 similar compounds. Some representative compounds for the major clusters are presented around the map.

To intuitively illustrate the structural space expansion of aerobic metabolism, we constructed a ‘structural cluster map’ consisting of anaerobic and aerobic metabolites. This map is a two-dimensional scatter plot that characterizes the structural similarity patterns of compounds. In this map, each compound is represented with a dot and the position of the dot is determined by its Tanimoto similarity to other compounds (based on shared substructural fragments) [27]. Similar compounds lie close to each other in the map and dissimilar structures are far from each other. Moreover, similar compounds (with Tanimoto similarity >0.85) [28], [29] pool together into clusters (the number of the clustered members is represented by the dot size). The cluster map for aerobic and anaerobic metabolites (Figure 3) shows that anaerobic compounds (represented by blue dots) dominate the lower part of the map while aerobic compounds (represented by red dots) occupy the central and upper part of the map representation. Clearly, this indicates that oxygen has expanded the structural space of metabolites considerably.

Analysis of the major clusters (big dots) of anaerobic and aerobic metabolites (Figure 3) shows that the anaerobic molecules mainly comprise of scaffolds of amino acids, pyrimidine and purine nucleotides, saccharides, glycosyl phosphates and folic acids, which are nearly all primary metabolites and are essential for core cellular functions. In comparison, the aerobic molecules mainly involve steroids, diterpenoids (*e.g.*, gibberellins), polyphenols (*e.g.*, flavonols and phenylpropanoids), alkaloids (*e.g.*, berberines) and macrocyclic lactones (*e.g.*, avermectins), most of which are secondary metabolites and are important for aspects of life that are known to be evolutionarily derived, such as transmembrane export and import (steroids), signal transfer (steroids, diterpenoids and polyphenols), defense against biotic factors (alkaloids and macrocyclic lactones) and organism protection from oxidation (polyphenols).

### The impact of oxygen on metabolic evolution in chemical space

To explore the impact of oxygen on metabolic evolution in chemical space, some commonly used chemical property descriptors were calculated for anaerobic and aerobic metabolites (Table 2). Since property distribution patterns in the metabolite datasets are far from normal (Figure S2), both median and mean values for these descriptors were calculated.

**Table 2:** Median/mean values of chemical property descriptors for anaerobic and aerobic metabolites.

Descriptors	Characterization	Median/Mean		*P* value^d^
		Anaerobic metabolites (n = 1174)	Aerobic metabolites (n = 520)	
MW^a^	Molecular weight	214.70/310.72	289.38/317.69	0.52
AREA^a^	Total molecular surface area	425.95/545.21	508.90/549.64	0.74
VOL^a^	Total molecular volume	613.75/822.00	803.80/856.24	0.17
AtomCount^a^	Total atom count	26/36.48	36/43.23	2.95E-06
Carbons^b^	Carbon atom count	8/10.94	15/16.06	1.06E-23
Oxygens^b^	Oxygen atom count	6/7.24	4/5.38	1.08E-13
Nitrogens^b^	Nitrogen atom count	1/1.67	0/0.73	1.19E-24
Sulfurs^b^	Sulfur atom count	0/0.10	0/0.11	0.57
Phosphorus^b^	Phosphorus atom count	0/0.68	0/0.08	6.12E-56
AlogP98^c^	Logarithm of partition coefficient, atom-type value, using latest parameters	−1.08/−0.80	1.26/1.43	2.51E-63
PSA^a^	Polar molecular surface area	242.40/280.12	169.47/195.99	9.96E-28
PV^a^	Polar molecular volume	170.50/247.37	127.61/165.27	1.71E-21
Acceptor^a^	H-bond acceptor count	6/7.66	4/5.56	1.21E-14
Donor^a^	H-bond donor count	3/3.81	2/2.37	8.83E-20
Hydrophobe^a^	Hydrophobic fragment count	1/1.43	2/2.45	3.09E-23
RingCount^a^	Ring count	1/1.34	3/2.55	3.11E-38
AromaticRings^b^	Aromatic ring count	0/0.44	0/0.79	3.40E-13
BondCount^a^	Total bond count	26/36.83	38/44.78	1.34E-07
RotBonds^a^	Rotatable bond count	7/8.97	5/6.22	4.99E-18
Chiral^a^	Chiral center count	2/3.02	2/3.78	4.69E-04

a calculated with Sybyl 7.0.

b calculated with Pipeline Pilot.

c calculated with Cerius2.

d *t*-test.

Although anaerobic and aerobic metabolites are similar in some bulk characteristics, such as molecular weight, total molecular surface area and total molecular volume, they are much different in other properties (Table 2). First, aerobic metabolites contain more total atoms than anaerobic compounds. Remarkably, aerobic metabolites possess more carbon atoms but less oxygen, nitrogen and phosphorus atoms than the latter. The low oxygen content in aerobic metabolites seems to violate intuition. However, the fact that most oxygen atoms in biological molecules do not come from molecular oxygen but from water and carbon dioxide help explain this anomaly. The high phosphorus content in anaerobic metabolites is in accordance with the prevalence of the phosphate group in these molecules [3]. Since oxygen, nitrogen and phosphorus usually form polar bonds with other atoms, while the bonds involving carbon atoms are always non-polar, we infer that the anaerobic metabolites are more polar than the aerobic compounds. This is supported by the fact that anaerobic metabolites have a lower logarithm of partition coefficient, larger polar molecular surface area and polar volume, more hydrogen-bond donors/acceptors, and less hydrophobic fragments and aromatic rings (Table 2). Second, it seems that anaerobic metabolites are from a conformational point of view more flexible than aerobic molecules, because the former have more rotatable bonds and contain less rings (including aromatic rings) than the latter.

**Figure 4:**
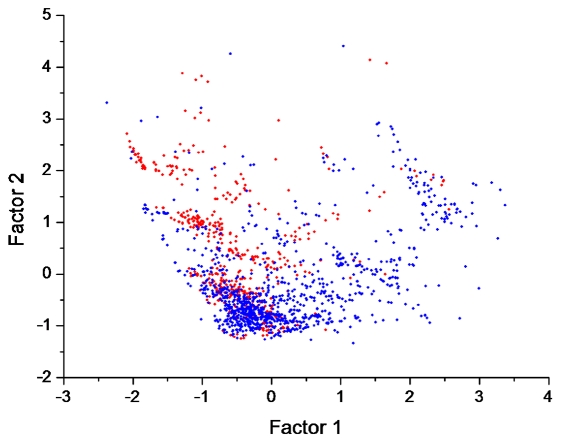
Chemical space of anaerobic and aerobic metabolites defined by the first two factors from an analysis of 20 descriptors. Aerobic metabolites (in blue) preferentially occupy the left and upper parts of the space, while anaerobic metabolites (in red) concentrate in the relatively lower part. Oxygen appears to have greatly helped metabolism to explore a wider chemical space.

To illustrate the difference of both kinds of metabolites in chemical space, factor analysis was performed on the chemical property descriptors. The first two factors, which can explain 78.6% of the variance, were extracted by principal component analysis and rotated by the Varimax method [30]. The distribution pattern of aerobic metabolites (in red) in the two-dimensional chemical space defined by these two factors clearly differs from that of anaerobic compounds (in blue) (Figure 4). Aerobic molecules occupy the relatively upper and left part of the space. Anaerobic molecules concentrate in the relatively lower part. From the Varimax normalized factor loadings (Table S3), we can find that the first factor explains 40.4% of the variance and contains high loadings (>0.85) from properties associated with polarity (*i.e.*, polar molecular surface area and polar volume, hydrogen bond acceptor count and oxygen atom count) (in bold). In comparison, the second factor, explaining 38.2% of the variance, contains important contributions (with loadings >0.85) from constitutional properties (*i.e.*, carbon atom count, hydrophobic fragment count, and total bond and atom count) (in bold, Table S3). These high-loaded descriptors embody major differences between anaerobic and aerobic metabolites (Table 2). We therefore conclude that oxygen has expanded the chemical space of metabolites considerably, mainly by enhancing the hydrophobicity and rigidity of metabolites. To test the robustness of this conclusion, we examined the latest KEGG version (60.0) and identified 486 aerobic reactions that use oxygen explicitly but were not available for simulation in Raymond and Segrè's dataset (Table S4). These reactions contain 440 products, in which 384 can be handled in calculation. Remarkably, the new aerobic metabolites show polarity (with an average AlogP98 of 2.18) and rigidity (with an average rotatable bond count of 5.92 and an average ring count of 2.08) that were similar to those previously recorded (Table 2). Therefore, our conclusions on chemical property features of aerobic metabolites are not significantly affected by KEGG expansion.

**Figure 5:**
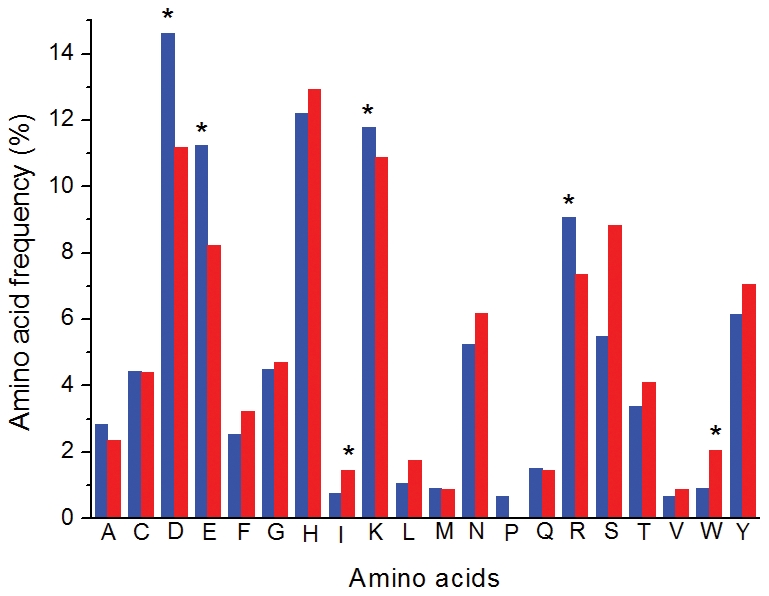
Amino acid compositions of catalytic sites in anaerobic (in blue) and aerobic (in red) enzymes. It can be seen that anaerobic enzymes use polar amino acid residues (*e.g.*, Asp, Glu, Lys and Arg) in catalytic sites more frequently (*P*<0.05), while aerobic enzymes use non-polar residues (*e.g.*, Trp and Ile) more often (*P*<0.05).

It is noteworthy and intriguing that differences in polarity of aerobic and anaerobic metabolites left imprints in residue compositions of corresponding enzymes. A search of the Catalytic Site Atlas (CSA) [31] revealed that the use of amino acid residues that exist in catalytic sites of 453 anaerobic and 257 aerobic enzymes was significantly biased. Remarkably, anaerobic enzymes use polar amino acid residues (*e.g.*, Asp, Glu, Lys and Arg) in catalytic sites more frequently (*P*<0.05), while aerobic enzymes use non-polar residues (*e.g.*, Trp and Ile) more often (*P*<0.05) (Figure 5). Patterns in polarity of catalytic residues therefore match patterns in polarity between aerobic and anaerobic metabolites.

**Figure 6:**
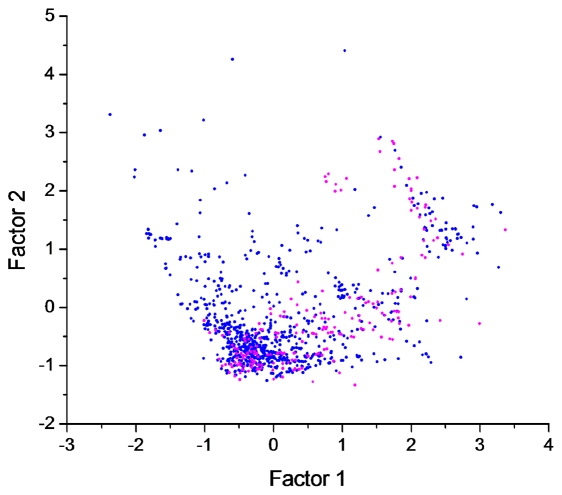
Chemical space of anaerobic metabolites defined by the first two factors from an analysis of 20 descriptors, showing early anaerobic metabolites (in magenta) preferentially occupying the right and lower parts of the space.

In evolution, metabolic networks expand as new chemistries and enzymes are made available to the cell [32]. We therefore explored how the chemical space of metabolites changed during the expansion of anaerobic networks. A large-scale phylogenomic analysis of metabolism based on analysis of protein domain structure identified the 163 most ancient enzymes (with relative ages ≤0.05 in a scale of 0 to 1, with 0 being the most ancient) [33]. From the metabolic reactions associated with these enzymes, we selected a set of 236 metabolites that are contained in the present anaerobic metabolite dataset, most of which are basic building blocks of life, such as amino acids, pyrimidine and purine nucleotides, saccharides, glycosyl phosphates and porphyrins. These compounds can be regarded as early appearing anaerobic metabolites and the other 938 should be considered late appearing anaerobic counterparts. Early metabolites occupy the right and lower part of the chemical space (Figure 6). The first factor is therefore able to discriminate metabolite age, indicating chemical differences between early and late metabolites reside in the properties associated with polarity. This is supported by a direct comparison between the twenty chemical property descriptors we analyzed, which also indicates that the early anaerobic metabolites are much more polar than their late counterparts (Table S5). The strong polarity of early anaerobic metabolites coincides with recent findings in the metabolome of yeast [34] that agree well with the widely accepted notion that the oceans are the cradle of life. Taken together, our results suggest that during metabolic evolution, metabolites in general got less and less polar, better meeting requirements to modulate membrane functions and to perform intercellular communications that exist in complex life [35].

### The impact of oxygen on evolution of metabolic reactions

The chemical features of aerobic metabolites raise an interesting question. Oxygenation reactions usually introduce hydroxyl groups and these groups are hydrogen donors/acceptors and are hydrophilic. Given this fact, how can we explain the observation that aerobic metabolites contain less hydrogen donors and acceptors and are more hydrophobic than anaerobic compounds? To address this question we investigated features that describe anaerobic and aerobic metabolic reactions.

Based on KEGG metabolic maps, 1,114 anaerobic reactions (covering 938 anaerobic metabolites) and 630 aerobic reactions (covering 480 aerobic metabolites) were identified as irreversible. During assignments, the “augmented reactions” defined in ref. 13 were not considered. We extracted 1342 anaerobic reaction pairs and 656 aerobic reaction pairs from these irreversible reactions, discarding enzyme cofactors and metabolites with molecular weights <70 Da. We then calculated net changes (from reactants to products) for some properties associated with polarity and averaged values (Table 3). Remarkably, products of anaerobic metabolic reactions tend to be less polar. This supports our previous observations that metabolite polarity reduced during the expansion of the anaerobic network. In contrast, the polarity variation trend in aerobic metabolic reactions is opposite to that in anaerobic reactions. This trend is compatible with the traits of oxygenation reactions, which usually add hydroxyl groups to products. However, the trend cannot explain why the aerobic metabolites are less polar than the anaerobic compounds. Considering the fact that the oxygen-dependent pathways largely start from the periphery of anaerobic metabolic network, whereas the anaerobic reactions tend to start from the center of the network (as above stated) [13], we speculate that the answer to this question may lie in the different starting points of aerobic and anaerobic metabolic reactions.

**Table 3:** Averaged net changes (from reactants to products) of some properties associated with polarity for anaerobic and aerobic reaction pairs.

Descriptors	Characterization	Averaged net changes	
		Anaerobic reaction pair	Aerobic reaction pair
AlogP98^a^	Logarithm of partition coefficient, atom-type value, using latest parameters	0.31	−0.14
PSA^b^	Polar molecular surface area	−20.85	7.12
PV^b^	Polar molecular volume	−25.82	5.95
Acceptor^b^	H-bond acceptor count	−0.82	0.42
Donor^b^	H-bond donor count	−0.43	0.18

a calculated with Cerius2.

b calculated with Sybyl 7.0.

**Figure 7:**
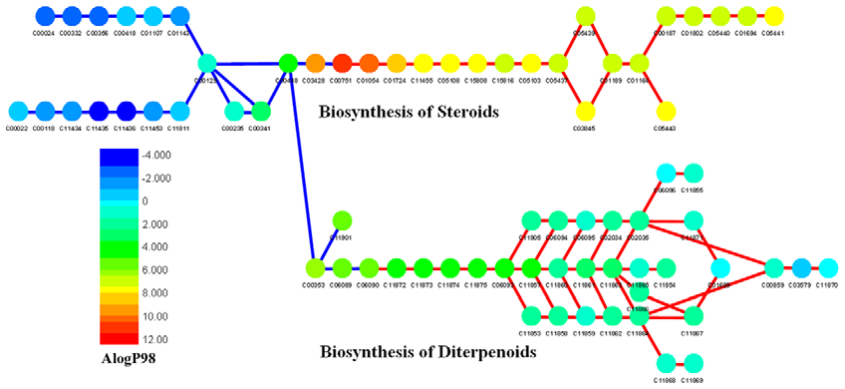
Polarity variation trends in pathways for biosynthesis of steroids and diterpenoids. Each metabolite is represented with a dot. Blue lines connect anaerobic metabolites (left) and red lines connect aerobic metabolites (right). The metabolite polarity descriptor (AlogP98) is indicated by different colors, from blue (strong polar) to red (strong non-polar). The polarity of metabolites decreases steadily to the minimum with the progression of anaerobic reactions (from left to right). In contrast, the polarity of aerobic metabolites increases with the expansion of aerobic networks but is on average still weaker than that of anaerobic metabolites.

Our calculation shows that the average logarithm of the partition coefficient (AlogP98) of the initial anaerobic reactants (−1.63) (Table S1) is much lower than that of the initial aerobic reactants (2.75) (Table S2). This suggests aerobic metabolites are less polar because of the hydrophobic starting points. This explanation is clearly represented by the polarity variation trends in pathways for biosynthesis of steroids and diterpenoids, two representatives of aerobic modules (Figure 7). The figure shows that with the progression of anaerobic reactions, the polarity of metabolites decreases steadily to the minimum (with AlogP98 of 11.33). In contrast, the polarity of aerobic metabolites increases (with an average AlogP98 of 4.05) with the expansion of aerobic networks but is still weaker than that of anaerobic metabolites (with an average AlogP98 of 1.31).

In summary, the present chemoinformatic analysis reveals two major impacts of oxygen on metabolic evolution that unfold in chemical space. First, the new reaction types of aerobic metabolism embody a large number of novel molecular scaffolds. In particular, the epoxidation of squalene by oxygen and subsequent oxygen-dependent reactions generate a series of steroids, which are most popular scaffolds in aerobic metabolites. Since the hydroxyl group is directly attached to the ring structure at C3, the polar layout of steroids contrasts with their anaerobic counterparts, the hopanoids. This layout enables steroids to play crucial roles in endo- and exocytosis of unicellular and multicellular eukaryotes [36]. Since complex organisms depend largely on sophisticated transmembrane export and import processes, the invention of steroids by aerobic metabolism represents a key step in the prokaryote-to-eukaryote transition responsible for multicellularity and higher organisms [37]. Second, aerobic metabolic reactions start from strong hydrophobic substrates and aerobic metabolites are on average less polar than anaerobic compounds. This makes aerobic metabolites better fit to traverse membranes and to serve as nuclear receptor ligands [35]. These ligands are part of the nuclear signaling system, which is critical to the functioning of complex organisms. Although some oxygen-dependent reactions could occur without oxygen, aerobic reactions are thermodynamically more efficient than the anaerobic counterparts [15]. This is of special importance for the reactions beginning with hydrophobic molecules, because hydrophobic metabolites have relatively low cellular concentrations and thus are less bioavailable [18], [34]. Taken together, it can be concluded that oxygen enabled metabolism to explore a wider structural and chemical space in an efficient manner, which is definitely helpful to enhance the complexity of cellular organization.

## Methods

### Data handling

The metabolic networks simulated by Raymond and Segrè (http://prelude.bu.edu/O2/networks.html) consist of 1,326 anaerobic metabolites (blue nodes) and 538 aerobic metabolites (red nodes) [13]. Out of these, we collected 1,174 anaerobic metabolites and 520 aerobic metabolites with clearly defined structures (without R group or polymeric form) as follows. First, for multi-component records, the small fragments (counter-ions in salts, solvent molecules) were removed and only the largest fragments were retained. Second, hydrogen atoms were added to fill the valences of heavy atoms and to neutralize the molecular charges. Finally, 3D structures were generated for all of the compounds. These operations were performed with Pipeline Pilot (Version 8.5, SciTegic Accelrys Inc. San Diego, CA.).

### Structural and chemical property calculations

Molecular scaffolds were generated with the Murcko method [38]. Scaffolds are defined as contiguous ring systems plus chains that link them, and were identified using “Generate Fragments” components in Pipeline Pilot, during which extra-cyclic double bonds and linker double bonds were kept.

Tanimoto coefficient (TC) has been widely used to characterize the structural similarity of molecules [27]–[29]. TC is defined as follows:
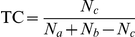
where *N*_a_ and *N*_b_ are the number of bits set for binary fingerprints of molecules A and B, respectively, and *N*_c_ is the set bits that A and B have in common. The structural cluster map was generated with Benchware DataMiner (Version 1.6. Tripos Associates Inc. St. Louis, MO.), adopting default parameters. The plotting procedure is as follows. First, we conducted a principal component analysis of substructural fingerprints (UNITY 2D fragments embedded in Benchware DataMiner) and used the first two components as the initial coordinates for the first batch of compounds (randomly selected from the dataset). Second, all compounds were added to the plot of already projected compounds using Tanimoto similarities of shared UNITY 2D substructural fragments. Similar compounds (with Tanimoto similarity >0.85) were pooled together into clusters. Finally, the distances were collapsed over the horizon. The non-linear mapping method (NLM) was used to minimize the overall fractional error and preserve the actual distances in many dimensions when plotting in fewer dimensions.

The commonly used chemical property descriptors, including Molecular weight, Total molecular surface area, Total molecular volume, Total atom count, Polar molecular surface area, Polar molecular volume, H-bond acceptor count, H-bond donor count, Hydrophobic fragment count, Ring count, Total bond count, Rotatable bond count and Chiral center count, were calculated with Sybyl 7.0. Carbon atom count, Oxygen atom count, Nitrogen atom count, Sulfur atom count, Phosphorus atom count and Aromatic ring count were calculated by Pipeline Pilot. Logarithm of partition coefficient (AlogP98) was calculated by Cerius 2 (Version 4.11L, Accelrys Inc. San Diego, CA.). All of the statistical analyses were performed with SPSS (Version 15.0, SPSS Inc. Chicago, IL.).

## Supporting Information

Figure S1 Illustration of anaerobic and aerobic metabolic networks.
(DOC)

Figure S2 Chemical property distribution patterns in the anaerobic and aerobic metabolite datasets.
(DOC)

Table S1 Basic information for the 48 major anaerobic modules.
(DOC)

Table S2 Basic information for the 19 major aerobic modules.
(DOC)

Table S3 Descriptors of chemical space consisting of anaerobic and aerobic metabolites and corresponding loadings (Varimax normalized) for the first two factors.
(DOC)

Table S4 Basic information for the additional aerobic metabolic reactions.
(DOC)

Table S5 Property comparison between early and late anaerobic metabolites.
(DOC)
