# Supplementary material for: The Impact of Oxygen on Metabolic Evolution: A Chemoinformatic Investigation
Source: PLoS Comput Biol. 2012 Mar 15;8(3):e1002426. doi: 10.1371/journal.pcbi.1002426 (PMC3305344; doi:10.1371/journal.pcbi.1002426)
Supplement: Figure S1 — Illustration of anaerobic and aerobic metabolic networks. (DOC) [file pcbi.1002426.s001.doc]

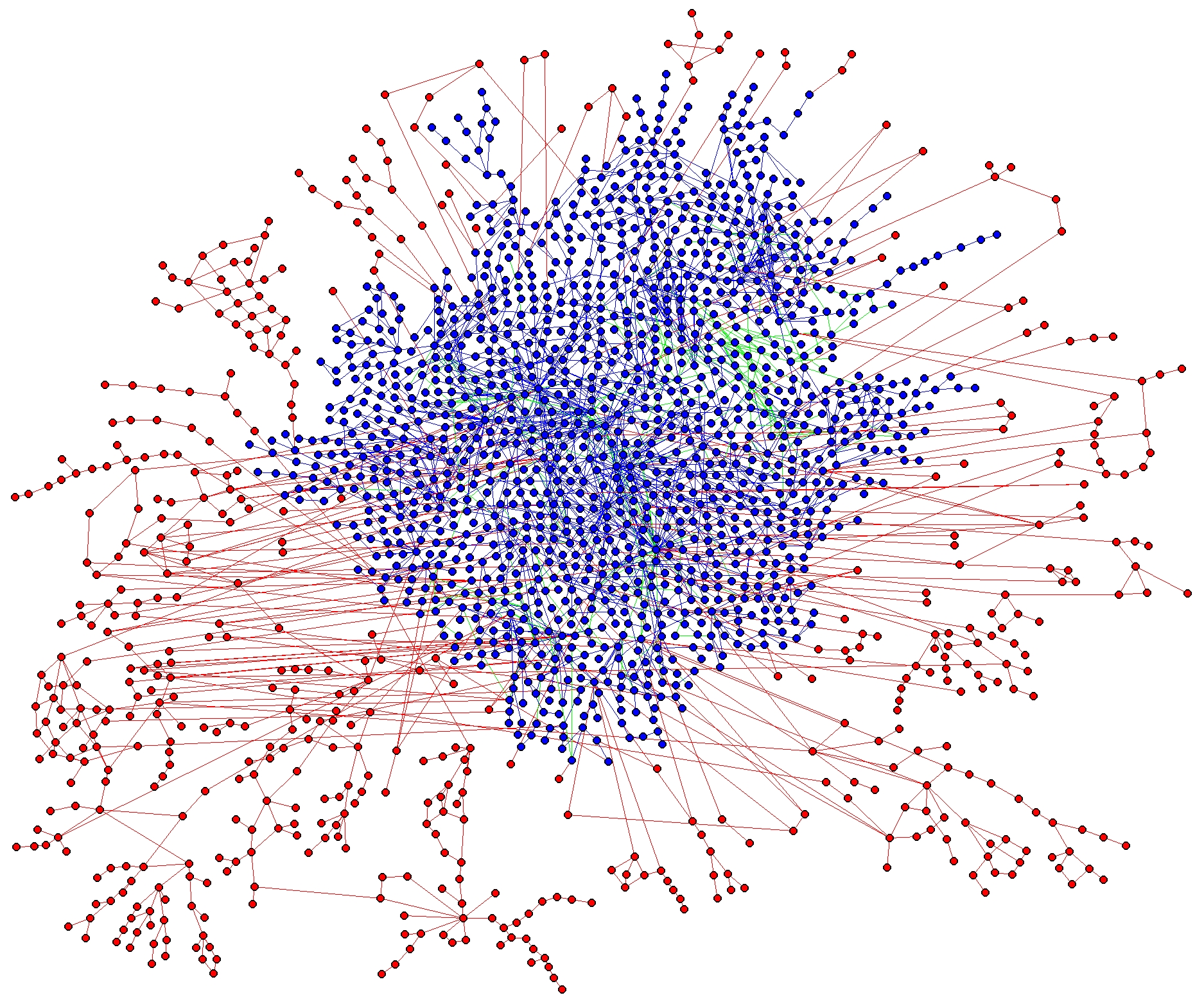


**Figure S1** Illustration of anaerobic and aerobic metabolic networks. It can be seen that aerobic metabolic reactions (in red) tend to start from the periphery of the anaerobic network (in blue) (from Raymond J, Segrè D, Science 2006, 311: 1764–1767).
