# Supplementary material for: The Impact of Oxygen on Metabolic Evolution: A Chemoinformatic Investigation
Source: PLoS Comput Biol. 2012 Mar 15;8(3):e1002426. doi: 10.1371/journal.pcbi.1002426 (PMC3305344; doi:10.1371/journal.pcbi.1002426)
Supplement: Figure S2 — Chemical property distribution patterns in the anaerobic and aerobic metabolite datasets. (DOC) [file pcbi.1002426.s002.doc]

**Fig. S2 (A) Fig. S2 (B)**

**Fig. S2 (C) Fig. S2 (D)**

**Fig. S2 (E) Fig. S2 (F)**

**Fig. S2 (G) Fig. S2 (H)**

**Fig. S2 (I) Fig. S2 (J)**

**Fig. S2 (K) Fig. S2 (L)**

**Fig. S2 (M) Fig. S2 (N)**

**Fig. S2 (O) Fig. S2 (P)**

**Fig. S2 (Q) Fig. S2 (R)**

**Fig. S2 (S) Fig. S2 (T)**

**Figure S2** Chemical property distribution patterns in the anaerobic (in blue) and aerobic (in red) metabolite datasets. (A) Molecular weight distribution pattern; (B) Total molecular surface area; (C) Total molecular volume; (D) Total atom count; (E) Carbon atom count; (F) Oxygen atom count; (G) Nitrogen atom count; (H) Sulfur atom count; (I) Phosphorus atom count; (J) AlogP98; (K) Polar molecular surface area; (L) Polar molecular volume; (M) H-bond acceptor count; (N) H-bond donor count; (O) Hydrophobic fragment count; (P) Ring count; (Q) Aromatic ring count; (R) Total bond count; (S) Rotatable bond count; (T) Chiral center count.
