# Supplementary material for: The Impact of Oxygen on Metabolic Evolution: A Chemoinformatic Investigation
Source: PLoS Comput Biol. 2012 Mar 15;8(3):e1002426. doi: 10.1371/journal.pcbi.1002426 (PMC3305344; doi:10.1371/journal.pcbi.1002426)
Supplement: Table S1 — Basic information for the 48 major anaerobic modules. (DOC) [file pcbi.1002426.s003.doc]

### Table S1 Basic information for the 48 major anaerobic modules.

| **Module**  **(Pathway in KEGG)** | **Metabolite count** | **Initial reactants**  **(reaction degree)** | **AlogP98** |
| --- | --- | --- | --- |
| purine metabolism | 61 | ITP (17)  GTP (15)  L-glutamine (17)  5-phosphoribosylamine (2)  PRPP (6) | -2.64  -2.03  -1.61  -2.18  -2.64 |
| pentose and glucuronate interconversions | 47 | UDP-glucuronate (5)  L-arabinose (7)  L-gulonate (4)  D-ribulose 5-phosphate (7)  D-fructose 6-phosphate (11) | -4.20  -2.00  -2.81  -2.25  -2.45 |
| pyrimidine metabolism | 39 | carbamoyl phosphate (3)  L-glutamine (17)  PRPP (6)  UDP-glucose (15)  UTP (9)  uracil (2) | -0.67  -1.61  -2.03  -4.33  -2.65  -0.82 |
| starch and sucrose metabolism | 39 | cyclomaltodextrin (1)  isomaltose (2)  celloheptaose (2) | -6.99  -4.26  -12.99 |
| arginine and proline metabolism | 38 | L-arginine (12) | -1.35 |
| glyoxylate and dicarboxylate metabolism | 38 | D-ribulose 1,5-bisphosphate (1)  formyl phosphate (1)  2-hydroxy-3-oxopropanoate (4)  hydroxypyruvate (5) | -2.13  -0.54  -1.02  -1.00 |
| fructose and mannose metabolism | 37 | sorbose 1-phosphate (1)  β-D-fructose 6-phosphate (11)  α-D-glucose (15) | -2.76  -2.45  -2.51 |
| benzoate degradation via hydroxylation | 36 | 4-hydroxybenzoate (4) | 1.22 |
| galactose metabolism | 36 | UDP-D-galactose (8)  α-D-glucose (15)  D-galactose (13) | -4.33  -2.51  -2.51 |
| glycine, serine and threonine metabolism | 35 | L-serine (14)  L-aspartate (16)  pyruvate (25) | -1.57  -1.30  -0.33 |
| fatty acid metabolism | 35 | acetyl-CoA (35) | -2.54 |
| urea cycle and metabolism of amino groups | 34 | L-glutamate (24)  L-arginine (12)  carbamoyl phosphate (3)  L-aspartate (16)  S-adenosyl-L-methionine (4)  putrescine (6) | -0.98  -1.35  -0.67  -1.30  -2.50  -0.83 |
| propanoate metabolism | 33 | succinyl-CoA (4)  L-lactate (7)  propanoyl-CoA (9)  2-hydroxybutyrate (1)  (S)-2-methylmalate (4)  1-aminocyclopropane-1-carboxylate (1) | -2.70  -0.39  -1.88  0.13  -0.70  -0.61 |
| butanoate metabolism | 32 | L-glutamate (24)  pyruvate (25)  2-oxoglutarate (11)  succinate semialdehyde (8) | -0.98  -0.33  -0.48  -0.34 |
| C_5_-branched dibasic acid metabolism | 32 | L-glutamate (24)  pyruvate (25) | -0.98  -0.33 |
| pentose phosphate pathway | 31 | α-D-glucose 6-phosphate (8)  β-D-glucose (5)  D-gluconate (4) | -2.40  -2.51  -2.81 |
| lysine degradation | 30 | L-lysine (11)  glutaryl-CoA (3) | -0.76  -2.24 |
| phenylalanine metabolism | 30 | L-phenylalanine (4) | 0.87 |
| ascorbate and aldarate metabolism | 30 | inositol (9)  UDP-glucose (15)  GDP-mannose (21)  D-galacturonate (4) | -3.06  -4.33  -4.45  -2.39 |
| nucleotide sugars metabolism | 28 | D-galacturonate (4)  UDP-D-galactose (8) | -2.39  -4.33 |
| lysine biosynthesis | 27 | L-aspartate (16)  acetyl-CoA (35) | -1.30  -2.54 |
| tyrosine metabolism | 27 | L-tyrosine (6)  4-hydroxyphenylacetate (3) | 0.63  1.25 |
| β-alanine metabolism | 27 | malonyl-CoA (11)  β-nitropropanoate (1)  β-aminopropionitrile (1)  L-aspartate (16)  spermine (1) | -2.73  0.02  -0.67  -1.30  -1.50 |
| polyketide sugar unit biosynthesis | 27 | D-glucose 1-phosphate (20) | -2.40 |
| pyruvate metabolism | 27 | methylglyoxal (6)  pyruvate (25)  phosphoenolpyruvate (19)  glycerone phosphate (14) | -0.31  -0.33  -0.30  -1.33 |
| benzoate degradation via CoA ligation | 27 | gallate (5)  anthranilate (6)  benzoate (6) | 0.73  0.71  1.46 |
| fatty acid elongation in mitochondria | 26 | acetyl-CoA (35)  butanoyl-CoA (11) | -2.54  -1.42 |
| glutamate metabolism | 25 | L-glutamine (17) | -1.61 |
| folate biosynthesis | 25 | GTP (15) | -2.77 |
| alkaloid biosynthesis II | 25 | L-lysine (11)  putrescine (6)  L-phenylalanine (4)  nicotinate (6) | -0.76  -0.83  0.87  0.31 |
| tryptophan metabolism | 24 | L-tryptophan (6) | 1.17 |
| phenylalanine, tyrosine and tryptophan biosynthesis | 23 | D-erythrose 4-phosphate (7)  phosphoenolpyruvate (19)  L-tyrosine (5) | -1.65  -0.30  0.63 |
| carbon fixation | 23 | 3-phospho-D-glycerate (7) | -1.17 |
| vitamin B6 metabolism | 23 | D-erythrose 4-phosphate (7)  D-ribulose 5-phosphate (7)  D-glyceraldehyde 3-phosphate (17) | -1.65  -2.25  -1.14 |
| glycolysis/gluconeogenesis | 22 | D-glucose (24)  D-glucose 1-phosphate (20) | -2.51  -2.40 |
| aminosugars metabolism | 22 | D-fructose 6-phosphate (11) | -2.45 |
| nicotinate and nicotinamide metabolism | 22 | L-aspartate (16) | -1.30 |
| biosynthesis of steroids | 20 | HMG-CoA (2)  D-glyceraldehyde 3-phosphate (17) | -3.00  -1.14 |
| alanine and aspartate metabolism | 20 | L-alanine (4)  L-aspartate (16) | -0.68  -1.30 |
| biosynthesis of 12-, 14- and 16-membered macrolides | 20 | propanoyl-CoA (9)  methylmalonyl-CoA (7)  malonyl-CoA (11) | -1.88  -2.27  -2.73 |
| toluene and xylene degradation | 20 | (R)-mandelate (3) | 0.84 |
| styrene degradation | 20 | styrene (1) | 2.38 |
| pantothenate and CoA biosynthesis | 20 | pyruvate (25)  uracil (2) | -0.33  -0.82 |
| terpenoid biosynthesis | 20 | isopentenyl diphosphate (5) | 0.89 |
| inositol phosphate metabolism | 19 | inositol 1-phosphate (2) | -2.95 |
| porphyrin and chlorophyll metabolism | 19 | L-glutamate (24)  glycine (11) | -0.98  -1.17 |
| Methionine metabolism | 18 | L-homoserine (5) | -1.51 |
| citrate cycle | 17 | pyruvate (25)  phosphoenolpyruvate (19)  acetyl-CoA (35) | -0.33  -0.30  -2.54 |
