# Supplementary material for: The Impact of Oxygen on Metabolic Evolution: A Chemoinformatic Investigation
Source: PLoS Comput Biol. 2012 Mar 15;8(3):e1002426. doi: 10.1371/journal.pcbi.1002426 (PMC3305344; doi:10.1371/journal.pcbi.1002426)
Supplement: Table S2 — Basic information for the 19 major aerobic modules. (DOC) [file pcbi.1002426.s004.doc]

**Table S2** Basic information for the 19 major aerobic modules.

| **Module**  **(Pathway in KEGG)** | **Metabolite count** | **Initial reactants**  **(reaction degree)** | **AlogP98** | **Distance from anaerobic central metabolite** |
| --- | --- | --- | --- | --- |
| tyrosine metabolism | 38 | 4-hydroxyphenylacetate (3) | 1.25 | 4 (L-tyrosine) |
| tryptophan metabolism | 38 | L-tryptophan (6) | 1.17 | 5 (L-tyrosine) |
| diterpenoid biosynthesis | 33 | ent-kaurene (4) | 5.63 | 9 (acetyl-CoA) |
| phenylpropanoid biosynthesis | 30 | 4-coumarate (4)  trans-cinnamate (6) | 1.69  1.93 | 2 (L-tyrosine)  6 (L-tyrosine) |
| androgen and estrogen metabolism | 29 | 17alpha,20alpha-dihydroxycholesterol (1) | 5.28 | 11 (acetyl-CoA) |
| biosynthesis of 12-, 14- and 16-membered macrolides | 26 | 6,8a-seco-6,8a-deoxy-5-oxoavermectin “2b” aglycone (1)  6,8a-seco-6,8a-deoxy-5-oxoavermectin “1b” ag (1) | 4.01  4.86 | 6 (pyruvate)  6 (pyruvate) |
| limonene and pinene degradation | 26 | (-)-limonene (2)  (+)-limonene (2) | 3.50  3.50 | 10 (acetyl-CoA)  10 (acetyl-CoA) |
| indole and ipecac alkaloid biosynthesis | 22 | deoxyloganin (2) | -0.72 | 10 (acetyl-CoA) |
| flavonoid biosynthesis | 21 | apigenin (4) | 2.41 | 11 (L-tyrosine) |
| bile acid biosynthesis | 20 | cholesterol (11) | 7.38 | 25 (acetyl-CoA) |
| flavone and flavonol biosynthesis | 20 | apigenin (4) | 2.41 | 11 (L-tyrosine) |
| alkaloid biosynthesis I | 20 | 4-hydroxyphenylacetaldehyde (4) | 1.27 | 4 (L-tyrosine) |
| biosynthesis of steroids | 18 | squalene (4) | 11.33 | 12 (acetyl-CoA) |
| C_21_-steroid hormone metabolism | 13 | cholesterol (11) | 7.38 | 25 (acetyl-CoA) |
| purine metabolism | 11 | urate (2) | -1.57 | 7 (ITP) |
| phenylalanine metabolism | 11 | trans-cinnamate (6)  phenylpyruvate (6) | 1.93  1.37 | 6 (L-tyrosine)  5 (L-tyrosine) |
| novobiocin biosynthesis | 11 | dTDP-5-dimethyl-L-lyxose (1)  3-dimethylallyl-4-hydroxybenzoate (2) | -2.02  3.07 | 6 (UDP-glucose)  5 (L-tyrosine) |
| anthocyanin biosynthesis | 11 | leucocyanidin (3)  leucodelphinidin (2) | 1.19  0.95 | 8 (L-tyrosine)  9 (L-tyrosine) |
| penicillin and cephalosporin biosynthesis | 9 | ACV (2) | -0.51 | 5 (L-lysine) |
