# Supplementary material for: The Impact of Oxygen on Metabolic Evolution: A Chemoinformatic Investigation
Source: PLoS Comput Biol. 2012 Mar 15;8(3):e1002426. doi: 10.1371/journal.pcbi.1002426 (PMC3305344; doi:10.1371/journal.pcbi.1002426)
Supplement: Table S3 — Descriptors of chemical space consisting of anaerobic and aerobic metabolites and corresponding loadings (Varimax normalized) for the first two factors. (DOC) [file pcbi.1002426.s005.doc]

**Table S3 Descriptors of chemical space consisting of anaerobic and aerobic metabolites and corresponding loadings (Varimax normalized) for the first two factors.**

| **Descriptors** | **Characterization** | **Factor loadings^d^** | |
| --- | --- | --- | --- |
|  |  | 1 | 2 |
| MW^a^ | Molecular weight | 0.642 | 0.761 |
| AREA^a^ | Total molecular surface area | 0.600 | 0.773 |
| VOL^a^ | Total molecular volume | 0.566 | 0.812 |
| AtomCount^a^ | Total atom count | 0.399 | **0.899** |
| Carbon^b^ | Carbon atom count | 0.163 | **0.971** |
| Oxygen^b^ | Oxygen atom count | **0.888** | 0.364 |
| Nitrogen^b^ | Nitrogen atom count | 0.644 | 0.307 |
| Sulfur^b^ | Sulfur atom count | 0.395 | 0.230 |
| Phosphorus^b^ | Phosphorus atom count | 0.802 | 0.201 |
| AlogP98^c^ | Logarithm of partition coefficient, atom-type value, using latest parameters | -0.745 | 0.504 |
| PSA^a^ | Polar molecular surface area | **0.949** | 0.184 |
| PV^a^ | Polar molecular volume | **0.931** | 0.318 |
| Acceptor^a^ | H-bond acceptor count | **0.897** | 0.395 |
| Donor^a^ | H-bond donor count | 0.841 | 0.284 |
| Hydrophobe^a^ | Hydrophobic fragment count | 0.033 | **0.923** |
| RingCount^a^ | Ring count | 0.135 | 0.822 |
| AromaticRings^b^ | Aromatic ring count | 0.245 | 0.424 |
| BondCount^a^ | Total bond count | 0.389 | **0.906** |
| RotBonds^a^ | Rotatable bond count | 0.790 | 0.472 |
| Chiral^a^ | Chiral center count | 0.341 | 0.607 |

^a^ calculated with Sybyl 7.0

^b^ calculated with Pipeline Pilot

^c^ calculated with Cerius2

^d^ significant loadings are boldfaced
