# Supplementary material for: The Impact of Oxygen on Metabolic Evolution: A Chemoinformatic Investigation
Source: PLoS Comput Biol. 2012 Mar 15;8(3):e1002426. doi: 10.1371/journal.pcbi.1002426 (PMC3305344; doi:10.1371/journal.pcbi.1002426)
Supplement: Table S4 — Basic information for the additional aerobic metabolic reactions. (DOC) [file pcbi.1002426.s006.doc]

Table S4 Basic information for the additional aerobic metabolic reactions.

| Reaction ID | Definition | Equation | Substrate | Product |
| --- | --- | --- | --- | --- |
| R06839 | 3-Chlorocatechol + Oxygen <=> 2-Chloro-cis,cis-muconate | C05618 + C00007 <=> C03572 | C05618 | C03572 |
| R06851 | p-Benzenediol + NADPH + H+ + Oxygen <=> Benzene-1,2,4-triol + NADP+ + H2O | C00530 + C00005 + C00080 + C00007 <=> C02814 + C00006 + C00001 | C00530 | C02814 |
| R06856 | Chlorobenzene + Oxygen + NADH + H+ <=> 3-Chloro-cis-1,2-dihydroxycyclohexa-3,5-diene + NAD+ | C06990 + C00007 + C00004 + C00080 <=> C12837 + C00003 | C06990 | C12837 |
| R06865 | 3-Hexaprenyl-4-hydroxybenzoate + Oxygen <=> 3-Hexaprenyl-4,5-dihydroxybenzoate | C13425 + C00007 <=> C05200 | C13425 | C05200 |
| R06883 | Bisphenol A + NADH + H+ + Oxygen <=> 1,2-Bis(4-hydroxyphenyl)-2-propanol + NAD+ + H2O | C13624 + C00004 + C00080 + C00007 <=> C13629 + C00003 + C00001 | C13624 | C13629 |
| R06884 | Bisphenol A + NADH + H+ + Oxygen <=> 2,2-Bis(4-hydroxyphenyl)-1-propanol + NAD+ + H2O | C13624 + C00004 + C00080 + C00007 <=> C13631 + C00003 + C00001 | C13624 | C13631 |
| R06886 | 4,4'-Dihydroxy-alpha-methylstilbene + Oxygen <=> 4-Hydroxybenzaldehyde + 4'-Hydroxyacetophenone | C13632 + C00007 <=> C00633 + C10700 | C13632 | C00633 |
| R06888 | 2,2-Bis(4-hydroxyphenyl)-1-propanol + NADH + H+ + Oxygen <=> 2,3-Bis(4-hydroxyphenyl)-1,2-propanediol + NAD+ + H2O | C13631 + C00004 + C00080 + C00007 <=> C13634 + C00003 + C00001 | C13631 | C13634 |
| R06889 | 2,3-Bis(4-hydroxyphenyl)-1,2-propanediol + Oxygen <=> 4-Hydroxyphenacyl alcohol + 4-Hydroxybenzoate | C13634 + C00007 <=> C13635 + C00156 | C13634 | C13635 |
| R06890 | 4-Ethylphenol + NADPH + H+ + Oxygen <=> 1-(4'-Hydroxyphenyl)ethanol + NADP+ + H2O | C13637 + C00005 + C00080 + C00007 <=> C13638 + C00006 + C00001 | C13637 | C13638 |
| R06892 | 4'-Hydroxyacetophenone + NADPH + H+ + Oxygen <=> 4-Hydroxyphenyl acetate + NADP+ + H2O | C10700 + C00005 + C00080 + C00007 <=> C13636 + C00006 + C00001 | C10700 | C13636 |
| R06909 | 1-Methylnaphthalene + Oxygen + NADH + H+ <=> cis-1,2-Dihydroxy-1,2-dihydro-8-methylnaphthalene + NAD+ | C14082 + C00007 + C00004 + C00080 <=> C14083 + C00003 | C14082 | C14083 |
| R06911 | 1,2-Dihydroxy-8-methylnaphthalene + Oxygen <=> 2-Hydroxy-8-methylchromene-2-carboxylate | C14084 + C00007 <=> C14085 | C14084 | C14085 |
| R06915 | 3-Methylsalicylate + Oxygen + NADH + H+ <=> 2,3-Dihydroxytoluene + NAD+ + CO2 + H2O | C14088 + C00007 + C00004 + C00080 <=> C02923 + C00003 + C00011 + C00001 | C14088 | C02923 |
| R06916 | 1-Methylnaphthalene + Oxygen + NADH + H+ <=> 1-Hydroxymethylnaphthalene + NAD+ + H2O | C14082 + C00007 + C00004 + C00080 <=> C14089 + C00003 + C00001 | C14082 | C14089 |
| R06919 | 1-Naphthoic acid + Oxygen + NADH + H+ <=> cis-1,2-Dihydroxy-1,2-dihydro-8-carboxynaphthalene + NAD+ | C14091 + C00007 + C00004 + C00080 <=> C14092 + C00003 | C14091 | C14092 |
| R06921 | 1,2-Dihydroxy-8-carboxynaphthalene + Oxygen <=> 2-Carboxy-2-hydroxy-8-carboxychromene | C14093 + C00007 <=> C14094 | C14093 | C14094 |
| R06924 | 3-Formylsalicylic acid + Oxygen + H2O <=> 2-Hydroxyisophthalic acid + H2O2 | C14096 + C00007 + C00001 <=> C14097 + C00027 | C14096 | C14097 |
| R06926 | 2-Methylnaphthalene + Oxygen + NADH + H+ <=> (2-Naphthyl)methanol + NAD+ + H2O | C14098 + C00007 + C00004 + C00080 <=> C02909 + C00003 + C00001 | C14098 | C02909 |
| R06930 | (2-Naphthyl)methanol + Oxygen + NADH + H+ <=> cis-1,2-Dihydroxy-1,2-dihydro-7-hydroxymethylnaphthalene + NAD+ | C02909 + C00007 + C00004 + C00080 <=> C14104 + C00003 | C02909 | C14104 |
| R06932 | 1,2-Dihydroxy-7-hydroxymethylnaphthalene + Oxygen <=> 2-Hydroxy-7-hydroxymethylchromene-2-carboxylate | C14105 + C00007 <=> C14106 | C14105 | C14106 |
| R06936 | 4-Hydroxymethylsalicylate + Oxygen + NADH + H+ <=> 4-Hydroxymethylcatechol + NAD+ + H2O + CO2 | C14109 + C00007 + C00004 + C00080 <=> C14110 + C00003 + C00001 + C00011 | C14109 | C14110 |
| R06937 | 2-Methylnaphthalene + Oxygen + NADH + H+ <=> cis-1,2-Dihydroxy-1,2-dihydro-7-methylnaphthalene + NAD+ | C14098 + C00007 + C00004 + C00080 <=> C14102 + C00003 | C14098 | C14102 |
| R06939 | 4-Methylsalicylate + Oxygen + NADH + H+ <=> 4-Methylcatechol + NAD+ + H2O + CO2 | C14103 + C00007 + C00004 + C00080 <=> C06730 + C00003 + C00001 + C00011 | C14103 | C06730 |
| R06945 | Cyclohexane + Oxygen + NADH + H+ <=> Cyclohexanol + NAD+ + H2O | C11249 + C00007 + C00004 + C00080 <=> C00854 + C00003 + C00001 | C11249 | C00854 |
| R06946 | Zeaxanthin + NADH + H+ + Oxygen <=> Antheraxanthin + NAD+ + H2O | C06098 + C00004 + C00080 + C00007 <=> C08579 + C00003 + C00001 | C06098 | C08579 |
| R06947 | Antheraxanthin + NADH + H+ + Oxygen <=> Violaxanthin + NAD+ + H2O | C08579 + C00004 + C00080 + C00007 <=> C08614 + C00003 + C00001 | C08579 | C08614 |
| R06952 | 9'-cis-Neoxanthin + Oxygen <=> Xanthoxin + C25-Allenic-apo-aldehyde | C13431 + C00007 <=> C13453 + C14044 | C13431 | C13453 |
| R06953 | 9-cis-Violaxanthin + Oxygen <=> Xanthoxin + C25-Epoxy-apo-aldehyde | C13433 + C00007 <=> C13453 + C14045 | C13433 | C13453 |
| R06957 | Abscisic aldehyde + H2O + Oxygen <=> Abscisate + H2O2 | C13455 + C00001 + C00007 <=> C06082 + C00027 | C13455 | C06082 |
| R07000 | Naphthalene + NADPH + Oxygen + H+ <=> (1R,2S)-Naphthalene 1,2-oxide + NADP+ + H2O | C00829 + C00005 + C00007 + C00080 <=> C14786 + C00006 + C00001 | C00829 | C14786 |
| R07001 | Naphthalene + NADPH + Oxygen + H+ <=> (1S,2R)-Naphthalene 1,2-oxide + NADP+ + H2O | C00829 + C00005 + C00007 + C00080 <=> C14787 + C00006 + C00001 | C00829 | C14787 |
| R07016 | 1,2-Dihydronaphthalene-1,2-diol + NADPH + Oxygen + H+ <=> 1,2-Dihydroxy-3,4-epoxy-1,2,3,4-tetrahydronaphthalene + NADP+ + H2O | C06205 + C00005 + C00007 + C00080 <=> C14784 + C00006 + C00001 | C06205 | C14784 |
| R07020 | 2-Naphthol + NADPH + Oxygen + H+ <=> Naphthalene-1,2-diol + NADP+ + H2O | C11713 + C00005 + C00007 + C00080 <=> C03012 + C00006 + C00001 | C11713 | C03012 |
| R07021 | 1-Nitronaphthalene + NADPH + Oxygen + H+ <=> 1-Nitronaphthalene-7,8-oxide + NADP+ + H2O | C14040 + C00005 + C00007 + C00080 <=> C14802 + C00006 + C00001 | C14040 | C14802 |
| R07022 | 1-Nitronaphthalene + NADPH + Oxygen + H+ <=> 1-Nitronaphthalene-5,6-oxide + NADP+ + H2O | C14040 + C00005 + C00007 + C00080 <=> C14800 + C00006 + C00001 | C14040 | C14800 |
| R07038 | (5Z,8Z,11Z,14Z)-Icosatetraenoic acid + Oxygen <=> 12(R)-HPETE | C00219 + C00007 <=> C14812 | C00219 | C14812 |
| R07041 | (5Z,8Z,11Z,14Z)-Icosatetraenoic acid + Oxygen + NADPH + H+ <=> 20-HETE + NADP+ + H2O | C00219 + C00007 + C00005 + C00080 <=> C14748 + C00006 + C00001 | C00219 | C14748 |
| R07046 | (5Z,8Z,11Z,14Z)-Icosatetraenoic acid + Oxygen + NADPH + H+ <=> 19(S)-HETE + NADP+ + H2O | C00219 + C00007 + C00005 + C00080 <=> C14749 + C00006 + C00001 | C00219 | C14749 |
| R07047 | (5Z,8Z,11Z,14Z)-Icosatetraenoic acid + Oxygen <=> 11(R)-HPETE | C00219 + C00007 <=> C14820 | C00219 | C14820 |
| R07048 | (5Z,8Z,11Z,14Z)-Icosatetraenoic acid + Oxygen + NADPH + H+ <=> 14,15-EET + NADP+ + H2O | C00219 + C00007 + C00005 + C00080 <=> C14771 + C00006 + C00001 | C00219 | C14771 |
| R07049 | (5Z,8Z,11Z,14Z)-Icosatetraenoic acid + Oxygen <=> 9(S)-HPETE | C00219 + C00007 <=> C14821 | C00219 | C14821 |
| R07050 | (5Z,8Z,11Z,14Z)-Icosatetraenoic acid + Oxygen + NADPH + H+ <=> 11,12-EET + NADP+ + H2O | C00219 + C00007 + C00005 + C00080 <=> C14770 + C00006 + C00001 | C00219 | C14770 |
| R07051 | (5Z,8Z,11Z,14Z)-Icosatetraenoic acid + Oxygen + NADPH + H+ <=> 8,9-EET + NADP+ + H2O | C00219 + C00007 + C00005 + C00080 <=> C14769 + C00006 + C00001 | C00219 | C14769 |
| R07052 | (5Z,8Z,11Z,14Z)-Icosatetraenoic acid + Oxygen + NADPH + H+ <=> 5,6-EET + NADP+ + H2O | C00219 + C00007 + C00005 + C00080 <=> C14768 + C00006 + C00001 | C00219 | C14768 |
| R07053 | (5Z,8Z,11Z,14Z)-Icosatetraenoic acid + Oxygen <=> 8(S)-HPETE | C00219 + C00007 <=> C14823 | C00219 | C14823 |
| R07054 | (5Z,8Z,11Z,14Z)-Icosatetraenoic acid + Oxygen + NADPH + H+ <=> 16(R)-HETE + NADP+ + H2O | C00219 + C00007 + C00005 + C00080 <=> C14778 + C00006 + C00001 | C00219 | C14778 |
| R07055 | Linoleate + Oxygen + NADPH + H+ <=> 9(10)-EpOME + NADP+ + H2O | C01595 + C00007 + C00005 + C00080 <=> C14825 + C00006 + C00001 | C01595 | C14825 |
| R07056 | Linoleate + Oxygen + NADPH + H+ <=> 12(13)-EpOME + NADP+ + H2O | C01595 + C00007 + C00005 + C00080 <=> C14826 + C00006 + C00001 | C01595 | C14826 |
| R07057 | Linoleate + Oxygen <=> 9(S)-HPODE | C01595 + C00007 <=> C14827 | C01595 | C14827 |
| R07061 | Linoleate + Oxygen <=> 8(R)-HPODE | C01595 + C00007 <=> C14831 | C01595 | C14831 |
| R07063 | Linoleate + Reduced acceptor + Oxygen <=> (6Z,9Z,12Z)-Octadecatrienoic acid + Acceptor + 2 H2O | C01595 + C00030 + C00007 <=> C06426 + C00028 + 2 C00001 | C01595 | C06426 |
| R07066 | Bromobenzene + NADPH + Oxygen + H+ <=> Bromobenzene-3,4-oxide + NADP+ + H2O | C11036 + C00005 + C00007 + C00080 <=> C14839 + C00006 + C00001 | C11036 | C14839 |
| R07068 | Bromobenzene + NADPH + Oxygen + H+ <=> Bromobenzene-2,3-oxide + NADP+ + H2O | C11036 + C00005 + C00007 + C00080 <=> C14840 + C00006 + C00001 | C11036 | C14840 |
| R07075 | 4-Bromophenol + NADPH + Oxygen + H+ <=> 4-Bromophenol-2,3-epoxide + NADP+ + H2O | C14453 + C00005 + C00007 + C00080 <=> C14845 + C00006 + C00001 | C14453 | C14845 |
| R07079 | Benzo[a]pyrene + NADPH + Oxygen + H+ <=> Benzo[a]pyrene-9,10-oxide + NADP+ + H2O | C07535 + C00005 + C00007 + C00080 <=> C14849 + C00006 + C00001 | C07535 | C14849 |
| R07080 | Benzo[a]pyrene + NADPH + Oxygen + H+ <=> Benzo[a]pyrene-7,8-oxide + NADP+ + H2O | C07535 + C00005 + C00007 + C00080 <=> C14850 + C00006 + C00001 | C07535 | C14850 |
| R07081 | Benzo[a]pyrene + NADPH + Oxygen + H+ <=> Benzo[a]pyrene-4,5-oxide + NADP+ + H2O | C07535 + C00005 + C00007 + C00080 <=> C14851 + C00006 + C00001 | C07535 | C14851 |
| R07085 | Benzo[a]pyrene-7,8-diol + NADPH + Oxygen + H+ <=> Benzo[a]pyrene-7,8-dihydrodiol-9,10-oxide + NADP+ + H2O | C14852 + C00005 + C00007 + C00080 <=> C14853 + C00006 + C00001 | C14852 | C14853 |
| R07087 | 9-Hydroxybenzo[a]pyrene + NADPH + Oxygen + H+ <=> 9-Hydroxybenzo[a]pyrene-4,5-oxide + NADP+ + H2O | C14556 + C00005 + C00007 + C00080 <=> C14854 + C00006 + C00001 | C14556 | C14854 |
| R07088 | 1,1-Dichloroethylene + NADPH + Oxygen + H+ <=> 2,2-Dichloroacetaldehyde + NADP+ + H2O | C14039 + C00005 + C00007 + C00080 <=> C14858 + C00006 + C00001 | C14039 | C14858 |
| R07089 | 1,1-Dichloroethylene + NADPH + Oxygen + H+ <=> 1,1-Dichloroethylene epoxide + NADP+ + H2O | C14039 + C00005 + C00007 + C00080 <=> C14857 + C00006 + C00001 | C14039 | C14857 |
| R07090 | 1,1-Dichloroethylene + NADPH + Oxygen + H+ <=> Chloroacetyl chloride + NADP+ + H2O | C14039 + C00005 + C00007 + C00080 <=> C14859 + C00006 + C00001 | C14039 | C14859 |
| R07098 | Trichloroethene + NADPH + Oxygen + H+ <=> TCE epoxide + NADP+ + H2O | C06790 + C00005 + C00007 + C00080 <=> C11148 + C00006 + C00001 | C06790 | C11148 |
| R07099 | Trichloroethene + NADPH + Oxygen + H+ <=> Chloral + NADP+ + H2O | C06790 + C00005 + C00007 + C00080 <=> C14866 + C00006 + C00001 | C06790 | C14866 |
| R07107 | Trichloroethanol + NADPH + Oxygen + H+ <=> Trichloroacetate + NADP+ + H2O | C07490 + C00005 + C00007 + C00080 <=> C11150 + C00006 + C00001 | C07490 | C11150 |
| R07112 | 1,2-Dibromoethane + NADPH + Oxygen + H+ <=> 2-Bromoacetaldehyde + NADP+ + H2O + Hydrobromic acid | C11088 + C00005 + C00007 + C00080 <=> C14870 + C00006 + C00001 + C13645 | C11088 | C14870 |
| R07128 | Oplophorus luciferin + Oxygen <=> Oxidized Oplophorus luciferin + hn + CO2 | C15037 + C00007 <=> C15038 + C00205 + C00011 | C15037 | C15038 |
| R07151 | L-2-Hydroxyphytanate + Oxygen <=> 2-Oxophytanate + H2O2 | C02982 + C00007 <=> C02117 + C00027 | C02982 | C02117 |
| R07152 | Xylitol + Oxygen <=> Xylose + H2O2 | C00379 + C00007 <=> C01394 + C00027 | C00379 | C01394 |
| R07170 | (R)-6-Hydroxynicotine + H2O + Oxygen <=> 6-Hydroxypseudooxynicotine + H2O2 | C03043 + C00001 + C00007 <=> C01297 + C00027 | C03043 | C01297 |
| R07171 | NADH + H+ + Oxygen <=> NAD+ + H2O2 | C00004 + C00080 + C00007 <=> C00003 + C00027 | C00004 |  |
| R07172 | NADPH + H+ + Oxygen <=> NADP+ + H2O2 | C00005 + C00080 + C00007 <=> C00006 + C00027 | C00005 |  |
| R07183 | Pentane-2,4-dione + Oxygen <=> Acetate + Methylglyoxal | C15499 + C00007 <=> C00033 + C00546 | C15499 | C00033 |
| R07184 | Gibberellin A44 + 2-Oxoglutarate + Oxygen <=> Gibberellin A19 + Succinate + CO2 | C12308 + C00026 + C00007 <=> C02034 + C00042 + C00011 | C12308 | C02034 |
| R07185 | 2'-Deoxymugineic acid + 2-Oxoglutarate + Oxygen <=> Mugineic acid + Succinate + CO2 | C15485 + C00026 + C00007 <=> C15500 + C00042 + C00011 | C15485 | C15500 |
| R07186 | Mugineic acid + 2-Oxoglutarate + Oxygen <=> 3-Epihydroxymugineic acid + Succinate + CO2 | C15500 + C00026 + C00007 <=> C15501 + C00042 + C00011 | C15500 | C15501 |
| R07187 | 2'-Deoxymugineic acid + 2-Oxoglutarate + Oxygen <=> 3-Epihydroxy-2'-deoxymugineic acid + Succinate + CO2 | C15485 + C00026 + C00007 <=> C15502 + C00042 + C00011 | C15485 | C15502 |
| R07188 | Benzoate + NADH + H+ + Oxygen <=> 1,6-Dihydroxycyclohexa-2,4-diene-1-carboxylate + NAD+ | C00180 + C00004 + C00080 + C00007 <=> C04634 + C00003 | C00180 | C04634 |
| R07191 | 2,3,5,6-Tetrachlorophenol + NADPH + H+ + Oxygen <=> Tetrachlorohydroquinone + NADP+ + H2O | C15505 + C00005 + C00080 + C00007 <=> C03434 + C00006 + C00001 | C15505 | C03434 |
| R07192 | Ketosteroid + NADPH + H+ + Oxygen <=> Steroid ester + NADP+ + H2O | C06479 + C00005 + C00080 + C00007 <=> C15506 + C00006 + C00001 | C06479 | C15506 |
| R07193 | Ketosteroid + NADPH + H+ + Oxygen <=> Steroid lactone + NADP+ + H2O | C06479 + C00005 + C00080 + C00007 <=> C15507 + C00006 + C00001 | C06479 | C15507 |
| R07194 | Propene + NADH + H+ + Oxygen <=> 1,2-Epoxypropane + NAD+ + H2O | C11505 + C00004 + C00080 + C00007 <=> C15508 + C00003 + C00001 | C11505 | C15508 |
| R07195 | 2-Dimethylallyl-(6aS,11aS)-3,6a,9-trihydroxypterocarpan + NADPH + H+ + Oxygen <=> (-)-Glyceollin II + NADP+ + 2 H2O | C15509 + C00005 + C00080 + C00007 <=> C10422 + C00006 + 2 C00001 | C15509 | C10422 |
| R07196 | 2-Dimethylallyl-(6aS,11aS)-3,6a,9-trihydroxypterocarpan + NADPH + H+ + Oxygen <=> Glyceollin III + NADP+ + 2 H2O | C15509 + C00005 + C00080 + C00007 <=> C15511 + C00006 + 2 C00001 | C15509 | C15511 |
| R07197 | 4-Dimethylallyl-(6aS,11aS)-3,6a,9-trihydroxypterocarpan + NADPH + H+ + Oxygen <=> (-)-Glyceollin I + NADP+ + 2 H2O | C15510 + C00005 + C00080 + C00007 <=> C01701 + C00006 + 2 C00001 | C15510 | C01701 |
| R07198 | Liquiritigenin + NADPH + H+ + Oxygen <=> Licodione + NADP+ + H2O | C09762 + C00005 + C00080 + C00007 <=> C01592 + C00006 + C00001 | C09762 | C01592 |
| R07199 | Zeaxanthin + NADPH + H+ + Oxygen <=> Antheraxanthin + NADP+ + H2O | C06098 + C00005 + C00080 + C00007 <=> C08579 + C00006 + C00001 | C06098 | C08579 |
| R07200 | Antheraxanthin + NADPH + H+ + Oxygen <=> Violaxanthin + NADP+ + H2O | C08579 + C00005 + C00080 + C00007 <=> C08614 + C00006 + C00001 | C08579 | C08614 |
| R07201 | Phenylacetone + NADPH + H+ + Oxygen <=> Benzyl acetate + NADP+ + H2O | C15512 + C00005 + C00080 + C00007 <=> C15513 + C00006 + C00001 | C15512 | C15513 |
| R07202 | Abscisate + NADPH + H+ + Oxygen <=> 8'-Hydroxyabscisate + NADP+ + H2O | C06082 + C00005 + C00080 + C00007 <=> C15514 + C00006 + C00001 | C06082 | C15514 |
| R07203 | 3alpha-Hydroxy-5beta-cholanate + NADPH + H+ + Oxygen <=> 6beta-Hydroxylithocholate + NADP+ + H2O | C03990 + C00005 + C00080 + C00007 <=> C15515 + C00006 + C00001 | C03990 | C15515 |
| R07204 | 3alpha,7alpha-Dihydroxy-5beta-cholestane + NADPH + H+ + Oxygen <=> 3alpha,7alpha,12alpha-Trihydroxy-5beta-cholestane + NADP+ + H2O | C05452 + C00005 + C00080 + C00007 <=> C05454 + C00006 + C00001 | C05452 | C05454 |
| R07205 | Taurochenodeoxycholate + NADPH + H+ + Oxygen <=> Taurohyocholate + NADP+ + H2O | C05465 + C00005 + C00080 + C00007 <=> C15516 + C00006 + C00001 | C05465 | C15516 |
| R07206 | 3alpha-Hydroxy-5beta-cholanate + NADPH + H+ + Oxygen <=> Hyodeoxycholate + NADP+ + H2O | C03990 + C00005 + C00080 + C00007 <=> C15517 + C00006 + C00001 | C03990 | C15517 |
| R07207 | Cholesterol + NADPH + H+ + Oxygen <=> Cerebrosterol + NADP+ + H2O | C00187 + C00005 + C00080 + C00007 <=> C13550 + C00006 + C00001 | C00187 | C13550 |
| R07208 | Cerebrosterol + NADPH + H+ + Oxygen <=> (24S)-Cholest-5-ene-3beta,7alpha,24-triol + NADP+ + H2O | C13550 + C00005 + C00080 + C00007 <=> C15518 + C00006 + C00001 | C13550 | C15518 |
| R07209 | Cholest-5-ene-3beta,25-diol + NADPH + H+ + Oxygen <=> Cholest-5-ene-3beta,7alpha,25-triol + NADP+ + H2O | C15519 + C00005 + C00080 + C00007 <=> C15520 + C00006 + C00001 | C15519 | C15520 |
| R07210 | Alkanesulfonate + Reduced FMN + Oxygen <=> Aldehyde + FMN + Sulfite + H2O | C15521 + C01847 + C00007 <=> C00071 + C00061 + C00094 + C00001 | C15521 | C00071 |
| R07211 | L-Phenylalanine + Tetrahydrobiopterin + Oxygen <=> L-Tyrosine + 4a-Hydroxytetrahydrobiopterin | C00079 + C00272 + C00007 <=> C00082 + C15522 | C00079 | C00082 |
| R07212 | L-Tyrosine + Tetrahydrobiopterin + Oxygen <=> 3,4-Dihydroxy-L-phenylalanine + 4a-Hydroxytetrahydrobiopterin | C00082 + C00272 + C00007 <=> C00355 + C15522 | C00082 | C00355 |
| R07213 | L-Tryptophan + Tetrahydrobiopterin + Oxygen <=> 5-Hydroxy-L-tryptophan + 4a-Hydroxytetrahydrobiopterin | C00078 + C00272 + C00007 <=> C00643 + C15522 | C00078 | C00643 |
| R07214 | 1-Aminocyclopropane-1-carboxylate + Ascorbate + Oxygen <=> Ethylene + Cyanide + Dehydroascorbate + CO2 + 2 H2O | C01234 + C00072 + C00007 <=> C06547 + C00177 + C05422 + C00011 + 2 C00001 | C01234 | C06547 |
| R07215 | 5alpha-Cholest-7-en-3beta-ol + NADPH + H+ + Oxygen <=> Cholesta-5,7-dien-3beta-ol + NADP+ + 2 H2O | C01189 + C00005 + C00080 + C00007 <=> C01164 + C00006 + 2 C00001 | C01189 | C01164 |
| R07216 | Tetradecanoyl-CoA + NADPH + H+ + Oxygen <=> (E)-11-Tetradecenoyl-CoA + NADP+ + 2 H2O | C02593 + C00005 + C00080 + C00007 <=> C06736 + C00006 + 2 C00001 | C02593 | C06736 |
| R07217 | Tetradecanoyl-CoA + NADPH + H+ + Oxygen <=> (Z)-11-Tetradecenoyl-CoA + NADP+ + 2 H2O | C02593 + C00005 + C00080 + C00007 <=> C06737 + C00006 + 2 C00001 | C02593 | C06737 |
| R07218 | Cholesterol + Reduced acceptor + Oxygen <=> Cholest-5-ene-3beta,25-diol + Acceptor + H2O | C00187 + C00030 + C00007 <=> C15519 + C00028 + C00001 | C00187 | C15519 |
| R07221 | 6-Hydroxynicotinate + H2O + Oxygen <=> 2,6-Dihydroxynicotinate + H2O2 | C01020 + C00001 + C00007 <=> C15523 + C00027 | C01020 | C15523 |
| R07224 | Naringenin chalcone + Oxygen <=> Aureusidin + H2O | C06561 + C00007 <=> C08576 + C00001 | C06561 | C08576 |
| R07225 | 2 Eriodictyol chalcone + Oxygen <=> 2 Aureusidin + 2 H2O | 2 C15525 + C00007 <=> 2 C08576 + 2 C00001 | C15525 | C08576 |
| R07329 | Flavanone + 2-Oxoglutarate + Oxygen <=> Dihydroflavonol + Succinate + CO2 | C00766 + C00026 + C00007 <=> C15570 + C00042 + C00011 | C00766 | C15570 |
| R07353 | 6-(2-Amino-2-carboxyethyl)-7,8-dioxo-1,2,3,4,7,8-hexahydroquinoline-2,4-dicarboxylate + 3 Oxygen <=> PQQ + 2 H2O2 + 2 H2O | C15599 + 3 C00007 <=> C00113 + 2 C00027 + 2 C00001 | C15599 | C00113 |
| R07356 | 2-Dimethylallyl-(6aS,11aS)-3,6a,9-trihydroxypterocarpan + NADPH + H+ + Oxygen <=> Glyceollin + NADP+ + 2 H2O | C15509 + C00005 + C00080 + C00007 <=> C15601 + C00006 + 2 C00001 | C15509 | C15601 |
| R07357 | 4-Dimethylallyl-(6aS,11aS)-3,6a,9-trihydroxypterocarpan + NADPH + H+ + Oxygen <=> Glyceollin + NADP+ + 2 H2O | C15510 + C00005 + C00080 + C00007 <=> C15601 + C00006 + 2 C00001 | C15510 | C15601 |
| R07360 | Prenyl-L-cysteine + Oxygen + H2O <=> Prenal + L-Cysteine + H2O2 | C06751 + C00007 + C00001 <=> C15604 + C00097 + C00027 | C06751 | C15604 |
| R07362 | D-Tryptophan + Oxygen <=> N-Formyl-D-kynurenine | C00525 + C00007 <=> C15605 | C00525 | C15605 |
| R07363 | 1,2-Dihydroxy-5-(methylthio)pent-1-en-3-one + Oxygen <=> 3-(Methylthio)propionic acid + Formate + CO | C15606 + C00007 <=> C08276 + C00058 + C00237 | C15606 | C08276 |
| R07364 | 1,2-Dihydroxy-5-(methylthio)pent-1-en-3-one + Oxygen <=> 4-Methylthio-2-oxobutanoic acid + Formate | C15606 + C00007 <=> C01180 + C00058 | C15606 | C01180 |
| R07365 | 4 Sulfur + 4 H2O + Oxygen <=> 2 Hydrogen sulfide + 2 HSO3- + 2 H+ | 4 C00087 + 4 C00001 + C00007 <=> 2 C00283 + 2 C11481 + 2 C00080 | C00087 | C00283 |
| R07366 | Leucocyanidin + 2-Oxoglutarate + Oxygen <=> cis-Dihydroquercetin + Succinate + CO2 + H2O | C05906 + C00026 + C00007 <=> C12316 + C00042 + C00011 + C00001 | C05906 | C12316 |
| R07367 | Flavanone + 2-Oxoglutarate + Oxygen <=> Flavone + Succinate + CO2 + H2O | C00766 + C00026 + C00007 <=> C15608 + C00042 + C00011 + C00001 | C00766 | C15608 |
| R07368 | Dihydroflavonol + 2-Oxoglutarate + Oxygen <=> Flavonol + Succinate + CO2 + H2O | C15570 + C00026 + C00007 <=> C01495 + C00042 + C00011 + C00001 | C15570 | C01495 |
| R07369 | Flavanone + NADPH + H+ + Oxygen <=> 3'-Hydroxyflavanone + NADP+ + H2O | C00766 + C00005 + C00080 + C00007 <=> C15204 + C00006 + C00001 | C00766 | C15204 |
| R07370 | 3'-Hydroxyflavanone + NADPH + H+ + Oxygen <=> 3',5'-Dihydroxyflavanone + NADP+ + H2O | C15204 + C00005 + C00080 + C00007 <=> C15609 + C00006 + C00001 | C15204 | C15609 |
| R07371 | Isoflavone + NADPH + H+ + Oxygen <=> 2'-Hydroxyisoflavone + NADP+ + H2O | C00799 + C00005 + C00080 + C00007 <=> C02921 + C00006 + C00001 | C00799 | C02921 |
| R07372 | Cholest-5-ene-3beta,26-diol + NADPH + H+ + Oxygen <=> 7alpha,27-Dihydroxycholesterol + NADP+ + H2O | C15610 + C00005 + C00080 + C00007 <=> C06341 + C00006 + C00001 | C15610 | C06341 |
| R07373 | Senecionine + NADPH + H+ + Oxygen <=> Senecionine N-oxide + NADP+ + H2O | C06176 + C00005 + C00080 + C00007 <=> C15612 + C00006 + C00001 | C06176 | C15612 |
| R07400 | Cephalosporin C + H2O + Oxygen <=> (7R)-7-(5-Carboxy-5-oxopentanoyl)aminocephalosporinate + NH3 + H2O2 | C00916 + C00001 + C00007 <=> C04712 + C00014 + C00027 | C00916 | C04712 |
| R07403 | Indole + NADPH + H+ + Oxygen <=> Indolin-2-one + NADP+ + H2O | C00463 + C00005 + C00080 + C00007 <=> C12312 + C00006 + C00001 | C00463 | C12312 |
| R07409 | Choline + Oxygen + 2 Reduced ferredoxin + 2 H+ <=> Betaine aldehyde + 2 H2O + 2 Oxidized ferredoxin | C00114 + C00007 + 2 C00138 + 2 C00080 <=> C00576 + 2 C00001 + 2 C00139 | C00114 | C00576 |
| R07415 | gamma-L-Glutamylputrescine + H2O + Oxygen <=> gamma-Glutamyl-gamma-aminobutyraldehyde + NH3 + H2O2 | C15699 + C00001 + C00007 <=> C15700 + C00014 + C00027 | C15699 | C15700 |
| R07421 | Indolin-2-one + NADPH + H+ + Oxygen <=> 3-Hydroxyindolin-2-one + NADP+ + H2O | C12312 + C00005 + C00080 + C00007 <=> C11130 + C00006 + C00001 | C12312 | C11130 |
| R07422 | 3-Hydroxyindolin-2-one + NADPH + H+ + Oxygen <=> HBOA + NADP+ + H2O | C11130 + C00005 + C00080 + C00007 <=> C15769 + C00006 + C00001 | C11130 | C15769 |
| R07423 | HBOA + NADPH + H+ + Oxygen <=> DIBOA + NADP+ + H2O | C15769 + C00005 + C00080 + C00007 <=> C15770 + C00006 + C00001 | C15769 | C15770 |
| R07430 | 6-Oxocampestanol + Oxygen + NADPH <=> Cathasterone + NADP+ + H2O | C15789 + C00007 + C00005 <=> C15790 + C00006 + C00001 | C15789 | C15790 |
| R07431 | Cathasterone + Oxygen + Reduced acceptor <=> Teasterone + H2O + Acceptor | C15790 + C00007 + C00030 <=> C15791 + C00001 + C00028 | C15790 | C15791 |
| R07440 | Ferulate + NADPH + H+ + Oxygen <=> 5-Hydroxyferulate + NADP+ + H2O | C01494 + C00005 + C00080 + C00007 <=> C05619 + C00006 + C00001 | C01494 | C05619 |
| R07445 | Campesterol + Oxygen + NADPH <=> 22alpha-Hydroxy-campesterol + NADP+ + H2O | C01789 + C00007 + C00005 <=> C15795 + C00006 + C00001 | C01789 | C15795 |
| R07448 | 6-Deoxocathasterone + Oxygen <=> 6-Deoxoteasterone + H2O | C15798 + C00007 <=> C15799 + C00001 | C15798 | C15799 |
| R07450 | 6-Deoxocastasterone + Oxygen <=> 6alpha-Hydroxy-castasterone + H2O | C15802 + C00007 <=> C15803 + C00001 | C15802 | C15803 |
| R07452 | Campest-4-en-3-one + Oxygen + NADPH <=> 22alpha-Hydroxy-campest-4-en-3-one + NADP+ + H2O | C15785 + C00007 + C00005 <=> C15796 + C00006 + C00001 | C15785 | C15796 |
| R07453 | 5alpha-Campestan-3-one + Oxygen + NADPH <=> 22alpha-Hydroxy-5alpha-campestan-3-one + NADP+ + H2O | C15786 + C00007 + C00005 <=> C15797 + C00006 + C00001 | C15786 | C15797 |
| R07454 | Campestanol + Oxygen + NADPH <=> 6-Deoxocathasterone + NADP+ + H2O | C15787 + C00007 + C00005 <=> C15798 + C00006 + C00001 | C15787 | C15798 |
| R07455 | 6-Deoxoteasterone + Oxygen <=> Teasterone + H2O | C15799 + C00007 <=> C15791 + C00001 | C15799 | C15791 |
| R07457 | 3-Dehydro-6-deoxoteasterone + Oxygen <=> 3-Dehydroteasterone + H2O | C15800 + C00007 <=> C15792 + C00001 | C15800 | C15792 |
| R07458 | 6-Deoxotyphasterol + Oxygen <=> Typhasterol + H2O | C15801 + C00007 <=> C15793 + C00001 | C15801 | C15793 |
| R07467 | Campestanol + Oxygen + Reduced acceptor <=> 6alpha-Hydroxycampestanol + H2O + Acceptor | C15787 + C00007 + C00030 <=> C15788 + C00001 + C00028 | C15787 | C15788 |
| R07470 | Typhasterol + Oxygen + Reduced acceptor <=> Castasterone + H2O + Acceptor | C15793 + C00007 + C00030 <=> C15794 + C00001 + C00028 | C15793 | C15794 |
| R07474 | 6-Deoxotyphasterol + Oxygen <=> 6-Deoxocastasterone + H2O | C15801 + C00007 <=> C15802 + C00001 | C15801 | C15802 |
| R07509 | 14-Demethyllanosterol + 3 NADPH + 3 H+ + 3 Oxygen <=> 4alpha-Methylzymosterol-4-carboxylate + 3 NADP+ + 4 H2O | C05108 + 3 C00005 + 3 C00080 + 3 C00007 <=> C15808 + 3 C00006 + 4 C00001 | C05108 | C15808 |
| R07514 | 2 6-Hydroxypseudooxynicotine + Oxygen <=> 2 2,6-Dihydroxypseudooxynicotine | 2 C01297 + C00007 <=> 2 C15986 | C01297 | C15986 |
| R07525 | Spirilloxanthin + Oxygen <=> 2-Ketospirilloxanthin + H2O | C15881 + C00007 <=> C15884 + C00001 | C15881 | C15884 |
| R07526 | 2-Ketospirilloxanthin + Oxygen <=> 2,2'-Diketospirilloxanthin + H2O | C15884 + C00007 <=> C15885 + C00001 | C15884 | C15885 |
| R07537 | Spheroidene + Oxygen <=> Spheroidenone + H2O | C15900 + C00007 <=> C15903 + C00001 | C15900 | C15903 |
| R07538 | Hydroxyspheroidene + Oxygen <=> Hydroxyspheroidenone + H2O | C15902 + C00007 <=> C15905 + C00001 | C15902 | C15905 |
| R07598 | L-Lysine + Oxygen + H2O <=> L-2-Aminoadipate 6-semialdehyde + H2O2 + NH3 | C00047 + C00007 + C00001 <=> C04076 + C00027 + C00014 | C00047 | C04076 |
| R07657 | 1-Hydroxy-2-naphthoate + Oxygen <=> (3Z)-4-(2-Carboxyphenyl)-2-oxobut-3-enoate | C03203 + C00007 <=> C16149 | C03203 | C16149 |
| R07663 | Toluene-4-sulfonate + Oxygen <=> 4-Cresol + Sulfate | C06677 + C00007 <=> C01468 + C00059 | C06677 | C01468 |
| R07665 | 3-Cresol + Oxygen + NADH + H+ <=> 3-Hydroxybenzyl alcohol + NAD+ + H2O | C01467 + C00007 + C00004 + C00080 <=> C03351 + C00003 + C00001 | C01467 | C03351 |
| R07682 | 1,6-Naphthalenedisulfonic acid + Oxygen <=> 1,2-Dihydroxynaphthalene-6-sulfonate + Sulfite | C16193 + C00007 <=> C16196 + C00094 | C16193 | C16196 |
| R07683 | 2,6-Naphthalenedisulfonic acid + Oxygen <=> 1,2-Dihydroxynaphthalene-6-sulfonate + Sulfite | C16194 + C00007 <=> C16196 + C00094 | C16194 | C16196 |
| R07684 | 1,2-Dihydroxynaphthalene-6-sulfonate + Oxygen <=> (Z)-4-(2-Hydroxy-5-sulfonatophenyl)-2-oxo-3-butenoate | C16196 + C00007 <=> C16198 | C16196 | C16198 |
| R07686 | 5-Sulfosalicylate + Oxygen <=> 2,5-Dihydroxybenzoate + Sulfate | C16199 + C00007 <=> C00628 + C00059 | C16199 | C00628 |
| R07687 | Anthracene + Oxygen + 2 H+ + 2 e- <=> Anthracene-9,10-dihydrodiol | C14315 + C00007 + 2 C00080 + 2 C05359 <=> C16205 | C14315 | C16205 |
| R07691 | 3-Hydroxy-2-naphthoate + Oxygen <=> 3-[6-(Carboxymethylene)cyclohexa-2,4-dien-1-ylidene]-2-oxopropanate | C16212 + C00007 <=> C16214 | C16212 | C16214 |
| R07697 | Phenylboronic acid + Oxygen <=> Phenol + Boric acid | C16200 + C00007 <=> C00146 + C12486 | C16200 | C00146 |
| R07698 | 1-Naphthalenesulfonic acid + Oxygen <=> Naphthalene-1,2-diol + Sulfite | C16201 + C00007 <=> C03012 + C00094 | C16201 | C03012 |
| R07699 | 2-Naphthalenesulfonic acid + Oxygen <=> Naphthalene-1,2-diol + Sulfite | C16202 + C00007 <=> C03012 + C00094 | C16202 | C03012 |
| R07700 | Aniline + Oxygen <=> Catechol + NH3 | C00292 + C00007 <=> C00090 + C00014 | C00292 | C00090 |
| R07701 | 1,2-Anthracenediol + Oxygen <=> 4-(3-Hydroxy-2-naphthyl)-2-oxobut-3-enoic acid | C16204 + C00007 <=> C16210 | C16204 | C16210 |
| R07702 | 1,2-Anthracenediol + Oxygen <=> 3-(2-Carboxyvinyl)naphthalene-2-carboxylic acid | C16204 + C00007 <=> C16209 | C16204 | C16209 |
| R07704 | Anthracene + Oxygen + NADH + H+ <=> Anthracene cis-1,2-dihydrodiol + NAD+ | C14315 + C00007 + C00004 + C00080 <=> C16203 + C00003 | C14315 | C16203 |
| R07706 | Nitrobenzene + Oxygen <=> Catechol + Nitrite | C06813 + C00007 <=> C00090 + C00088 | C06813 | C00090 |
| R07709 | Salicylate + NADH + Oxygen + H+ <=> 2,5-Dihydroxybenzoate + NAD+ + H2O | C00805 + C00004 + C00007 + C00080 <=> C00628 + C00003 + C00001 | C00805 | C00628 |
| R07710 | Salicylate + NADPH + Oxygen + H+ <=> 2,5-Dihydroxybenzoate + NADP+ + H2O | C00805 + C00005 + C00007 + C00080 <=> C00628 + C00006 + C00001 | C00805 | C00628 |
| R07711 | Liquiritigenin + NADPH + H+ + Oxygen <=> 6,7,4'-Trihydroxyflavanone + NADP+ + H2O | C09762 + C00005 + C00080 + C00007 <=> C16232 + C00006 + C00001 | C09762 | C16232 |
| R07714 | 7,4'-Dihydroxyflavone + 2 NADPH + 2 H+ + Oxygen <=> 2,7,4'-Trihydroxyisoflavanone + 2 NADP+ + H2O | C12123 + 2 C00005 + 2 C00080 + C00007 <=> C15567 + 2 C00006 + C00001 | C12123 | C15567 |
| R07715 | 6,7,4'-Trihydroxyflavanone + NADPH + H+ + Oxygen <=> 2,6,7,4'-Tetrahydroxyisoflavanone + NADP+ + H2O | C16232 + C00005 + C00080 + C00007 <=> C16233 + C00006 + C00001 | C16232 | C16233 |
| R07746 | Pseudobaptigenin + NADPH + H+ + Oxygen <=> 2',7-Dihydroxy-4',5'-methylenedioxyisoflavone + NADP+ + H2O | C10522 + C00005 + C00080 + C00007 <=> C16226 + C00006 + C00001 | C10522 | C16226 |
| R07776 | Biochanin A + Oxygen + NADPH + H+ <=> Pratensein + NADP+ + H2O | C00814 + C00007 + C00005 + C00080 <=> C10520 + C00006 + C00001 | C00814 | C10520 |
| R07777 | Liquiritigenin + NADPH + H+ + Oxygen <=> 2,7,4'-Trihydroxyisoflavanone + NADP+ + H2O | C09762 + C00005 + C00080 + C00007 <=> C15567 + C00006 + C00001 | C09762 | C15567 |
| R07778 | Naringenin + NADPH + H+ + Oxygen <=> 2-Hydroxy-2,3-dihydrogenistein + NADP+ + H2O | C00509 + C00005 + C00080 + C00007 <=> C12631 + C00006 + C00001 | C00509 | C12631 |
| R07779 | Bromoxynil + 2 NADPH + 2 H+ + Oxygen <=> 2,6-Dibromohydroquinone + 2 NADP+ + Hydrogen cyanide + H2O | C04178 + 2 C00005 + 2 C00080 + C00007 <=> C16248 + 2 C00006 + C01326 + C00001 | C04178 | C16248 |
| R07793 | 4-Chloroaniline + Oxygen + 2 H+ <=> 4-Chlorocatechol + NH3 | C14450 + C00007 + 2 C00080 <=> C02375 + C00014 | C14450 | C02375 |
| R07795 | 3-Sulfocatechol + Oxygen + H2O <=> 2-Hydroxymuconate + Sulfite | C06336 + C00007 + C00001 <=> C02501 + C00094 | C06336 | C02501 |
| R07799 | Fluoren-9-one + Oxygen + 2 H+ <=> 1-Hydro-1,1a-dihydroxy-9-fluorenone | C06712 + C00007 + 2 C00080 <=> C16262 | C06712 | C16262 |
| R07801 | 2,3-Dihydroxy-2'-carboxybiphenyl + Oxygen <=> 2-Hydroxy-6-oxo-6-(2-carboxyphenyl)-hexa-2,4-dienoate | C16263 + C00007 <=> C16264 | C16263 | C16264 |
| R07827 | 3-Chlorocatechol + Oxygen <=> 3-Chloro-2-hydroxymuconic semialdehyde | C05618 + C00007 <=> C16266 | C05618 | C16266 |
| R07836 | Thiobenzamide + Oxygen + NADPH + H+ <=> Thiobenzamide S-oxide + NADP+ + H2O | C16281 + C00007 + C00005 + C00080 <=> C16283 + C00006 + C00001 | C16281 | C16283 |
| R07837 | Thiobenzamide S-oxide + NADPH + H+ + Oxygen <=> Thiobenzamide S,S-dioxide + NADP+ + H2O | C16283 + C00005 + C00080 + C00007 <=> C16285 + C00006 + C00001 | C16283 | C16285 |
| R07861 | (9Z,12Z,15Z)-Octadecatrienoic acid + Reduced acceptor + Oxygen <=> Stearidonic acid + Acceptor + 2 H2O | C06427 + C00030 + C00007 <=> C16300 + C00028 + 2 C00001 | C06427 | C16300 |
| R07864 | (9Z,12Z,15Z)-Octadecatrienoic acid + Oxygen <=> 9(S)-HPOT | C06427 + C00007 <=> C16321 | C06427 | C16321 |
| R07869 | (9Z,12Z,15Z)-Octadecatrienoic acid + Oxygen <=> 13(S)-HPOT | C06427 + C00007 <=> C04785 | C06427 | C04785 |
| R07933 | (9Z,12Z,15Z)-Octadecatrienoyl-CoA + Reduced acceptor + Oxygen <=> (6Z,9Z,12Z,15Z)-Octadecatetraenoyl-CoA + Acceptor + 2 H2O | C16162 + C00030 + C00007 <=> C16163 + C00028 + 2 C00001 | C16162 | C16163 |
| R07939 | Caffeine + NADPH + Oxygen + H+ <=> 1,7-Dimethylxanthine + NADP+ + Formaldehyde + H2O | C07481 + C00005 + C00007 + C00080 <=> C13747 + C00006 + C00067 + C00001 | C07481 | C13747 |
| R07942 | 1-Methylxanthine + H2O + Oxygen <=> 1-Methyluric acid + H2O2 | C16358 + C00001 + C00007 <=> C16359 + C00027 | C16358 | C16359 |
| R07948 | 2 2,3,6-Trihydroxypyridine + 2 Oxygen <=> Blue pigment + 2 H2O2 | 2 C03458 + 2 C00007 <=> C16152 + 2 C00027 | C03458 | C16152 |
| R07949 | 2,3,6-Trihydroxypyridine + Oxygen + H+ <=> Maleamate + 2 H+ + CO2 | C03458 + C00007 + C00080 <=> C01596 + 2 C00080 + C00011 | C03458 | C01596 |
| R07954 | Caffeine + NADH + H+ + Oxygen <=> 1,7-Dimethylxanthine + NAD+ + Formaldehyde + H2O | C07481 + C00004 + C00080 + C00007 <=> C13747 + C00003 + C00067 + C00001 | C07481 | C13747 |
| R07955 | Caffeine + NADH + H+ + Oxygen <=> Theobromine + NAD+ + Formaldehyde + H2O | C07481 + C00004 + C00080 + C00007 <=> C07480 + C00003 + C00067 + C00001 | C07481 | C07480 |
| R07956 | Caffeine + NADPH + H+ + Oxygen <=> Theobromine + NADP+ + Formaldehyde + H2O | C07481 + C00005 + C00080 + C00007 <=> C07480 + C00006 + C00067 + C00001 | C07481 | C07480 |
| R07957 | 1,7-Dimethylxanthine + NADH + H+ + Oxygen <=> 7-Methylxanthine + NAD+ + Formaldehyde + H2O | C13747 + C00004 + C00080 + C00007 <=> C16353 + C00003 + C00067 + C00001 | C13747 | C16353 |
| R07958 | 1,7-Dimethylxanthine + NADPH + H+ + Oxygen <=> 7-Methylxanthine + NADP+ + Formaldehyde + H2O | C13747 + C00005 + C00080 + C00007 <=> C16353 + C00006 + C00067 + C00001 | C13747 | C16353 |
| R07959 | 1,7-Dimethylxanthine + NADH + H+ + Oxygen <=> 1-Methylxanthine + NAD+ + Formaldehyde + H2O | C13747 + C00004 + C00080 + C00007 <=> C16358 + C00003 + C00067 + C00001 | C13747 | C16358 |
| R07960 | 1,7-Dimethylxanthine + NADPH + H+ + Oxygen <=> 1-Methylxanthine + NADP+ + Formaldehyde + H2O | C13747 + C00005 + C00080 + C00007 <=> C16358 + C00006 + C00067 + C00001 | C13747 | C16358 |
| R07961 | Theobromine + NADH + H+ + Oxygen <=> 7-Methylxanthine + NAD+ + Formaldehyde + H2O | C07480 + C00004 + C00080 + C00007 <=> C16353 + C00003 + C00067 + C00001 | C07480 | C16353 |
| R07962 | Theobromine + NADPH + H+ + Oxygen <=> 7-Methylxanthine + NADP+ + Formaldehyde + H2O | C07480 + C00005 + C00080 + C00007 <=> C16353 + C00006 + C00067 + C00001 | C07480 | C16353 |
| R07963 | Theobromine + NADH + H+ + Oxygen <=> 3-Methylxanthine + NAD+ + Formaldehyde + H2O | C07480 + C00004 + C00080 + C00007 <=> C16357 + C00003 + C00067 + C00001 | C07480 | C16357 |
| R07964 | Theobromine + NADPH + H+ + Oxygen <=> 3-Methylxanthine + NADP+ + Formaldehyde + H2O | C07480 + C00005 + C00080 + C00007 <=> C16357 + C00006 + C00067 + C00001 | C07480 | C16357 |
| R07965 | 7-Methylxanthine + NADH + H+ + Oxygen <=> Xanthine + NAD+ + Formaldehyde + H2O | C16353 + C00004 + C00080 + C00007 <=> C00385 + C00003 + C00067 + C00001 | C16353 | C00385 |
| R07966 | 7-Methylxanthine + NADPH + H+ + Oxygen <=> Xanthine + NADP+ + Formaldehyde + H2O | C16353 + C00005 + C00080 + C00007 <=> C00385 + C00006 + C00067 + C00001 | C16353 | C00385 |
| R07967 | 3-Methylxanthine + NADH + H+ + Oxygen <=> Xanthine + NAD+ + Formaldehyde + H2O | C16357 + C00004 + C00080 + C00007 <=> C00385 + C00003 + C00067 + C00001 | C16357 | C00385 |
| R07968 | 3-Methylxanthine + NADPH + H+ + Oxygen <=> Xanthine + NADP+ + Formaldehyde + H2O | C16357 + C00005 + C00080 + C00007 <=> C00385 + C00006 + C00067 + C00001 | C16357 | C00385 |
| R07969 | 1-Methylxanthine + NADH + H+ + Oxygen <=> Xanthine + NAD+ + Formaldehyde + H2O | C16358 + C00004 + C00080 + C00007 <=> C00385 + C00003 + C00067 + C00001 | C16358 | C00385 |
| R07970 | 1-Methylxanthine + NADPH + H+ + Oxygen <=> Xanthine + NADP+ + Formaldehyde + H2O | C16358 + C00005 + C00080 + C00007 <=> C00385 + C00006 + C00067 + C00001 | C16358 | C00385 |
| R07971 | Caffeine + NADH + H+ + Oxygen <=> Theophylline + NAD+ + Formaldehyde + H2O | C07481 + C00004 + C00080 + C00007 <=> C07130 + C00003 + C00067 + C00001 | C07481 | C07130 |
| R07972 | Caffeine + NADPH + H+ + Oxygen <=> Theophylline + NADP+ + Formaldehyde + H2O | C07481 + C00005 + C00080 + C00007 <=> C07130 + C00006 + C00067 + C00001 | C07481 | C07130 |
| R07973 | Theophylline + NADH + H+ + Oxygen <=> 3-Methylxanthine + NAD+ + Formaldehyde + H2O | C07130 + C00004 + C00080 + C00007 <=> C16357 + C00003 + C00067 + C00001 | C07130 | C16357 |
| R07974 | Theophylline + NADPH + H+ + Oxygen <=> 3-Methylxanthine + NADP+ + Formaldehyde + H2O | C07130 + C00005 + C00080 + C00007 <=> C16357 + C00006 + C00067 + C00001 | C07130 | C16357 |
| R07975 | Theophylline + NADH + H+ + Oxygen <=> 1-Methylxanthine + NAD+ + Formaldehyde + H2O | C07130 + C00004 + C00080 + C00007 <=> C16358 + C00003 + C00067 + C00001 | C07130 | C16358 |
| R07976 | Theophylline + NADPH + H+ + Oxygen <=> 1-Methylxanthine + NADP+ + Formaldehyde + H2O | C07130 + C00005 + C00080 + C00007 <=> C16358 + C00006 + C00067 + C00001 | C07130 | C16358 |
| R07977 | 1,7-Dimethylxanthine + Oxygen + H2O <=> 1,7-Dimethyluric acid + H2O2 | C13747 + C00007 + C00001 <=> C16356 + C00027 | C13747 | C16356 |
| R07978 | Theobromine + H2O + Oxygen <=> 3,7-Dimethyluric acid + H2O2 | C07480 + C00001 + C00007 <=> C16360 + C00027 | C07480 | C16360 |
| R07979 | 7-Methylxanthine + Oxygen + H2O <=> 7-Methyluric acid + H2O2 | C16353 + C00007 + C00001 <=> C16355 + C00027 | C16353 | C16355 |
| R07980 | Caffeine + Oxygen + 2 H+ <=> 1,3,7-Trimethyluric acid + H2O | C07481 + C00007 + 2 C00080 <=> C16361 + C00001 | C07481 | C16361 |
| R07981 | 1,3,7-Trimethyluric acid + Oxygen + 2 H2O <=> 3,6,8-Trimethylallantoin + CO2 + H2O2 | C16361 + C00007 + 2 C00001 <=> C16362 + C00011 + C00027 | C16361 | C16362 |
| R07983 | 2,6-Dihydroxynicotinate + H2O + Oxygen <=> Maleamate + CO2 + Formate | C15523 + C00001 + C00007 <=> C01596 + C00011 + C00058 | C15523 | C01596 |
| R07993 | Pinocembrin + 2-Oxoglutarate + Oxygen <=> Pinobanksin + Succinate + CO2 | C09827 + C00026 + C00007 <=> C09826 + C00042 + C00011 | C09827 | C09826 |
| R07996 | Liquiritigenin + 2-Oxoglutarate + Oxygen <=> Garbanzol + Succinate + CO2 | C09762 + C00026 + C00007 <=> C09751 + C00042 + C00011 | C09762 | C09751 |
| R07997 | Butin + 2-Oxoglutarate + Oxygen <=> Fustin + Succinate + CO2 | C09614 + C00026 + C00007 <=> C01378 + C00042 + C00011 | C09614 | C01378 |
| R08002 | Liquiritigenin + NADPH + H+ + Oxygen <=> Butin + NADP+ + H2O | C09762 + C00005 + C00080 + C00007 <=> C09614 + C00006 + C00001 | C09762 | C09614 |
| R08005 | Eriodictyol chalcone + Oxygen <=> Bracteatin + H2O | C15525 + C00007 <=> C08577 + C00001 | C15525 | C08577 |
| R08008 | 2',4,4',6'-Tetrahydroxychalcone 4'-O-glucoside + Oxygen <=> Aureusidin 6-O-glucoside + H2O | C16407 + C00007 <=> C16409 + C00001 | C16407 | C16409 |
| R08009 | 2',3,4,4',6'-Peptahydroxychalcone 4'-O-glucoside + Oxygen <=> Bracteatin 6-O-glucoside + H2O | C16408 + C00007 <=> C16410 + C00001 | C16408 | C16410 |
| R08010 | 2 2',3,4,4',6'-Peptahydroxychalcone 4'-O-glucoside + Oxygen <=> 2 Aureusidin 6-O-glucoside + 2 H2O | 2 C16408 + C00007 <=> 2 C16409 + 2 C00001 | C16408 | C16409 |
| R08020 | 4 4-Hydroxylamino-2,6-dinitrotoluene + Oxygen <=> 2 2,2',6,6'-Tetranitro-4,4'-azoxytoluene + 4 H2O | 4 C16392 + C00007 <=> 2 C16413 + 4 C00001 | C16392 | C16413 |
| R08032 | Garbanzol + NADPH + H+ + Oxygen <=> Fustin + NADP+ + H2O | C09751 + C00005 + C00080 + C00007 <=> C01378 + C00006 + C00001 | C09751 | C01378 |
| R08037 | 2 4-Hydroxylamino-2,6-dinitrotoluene + 2 2-Hydroxylamino-4,6-dinitrotoluene + Oxygen <=> 2 2,4',6,6'-Tetranitro-2',4-azoxytoluene + 4 H2O | 2 C16392 + 2 C16393 + C00007 <=> 2 C16412 + 4 C00001 | C16392 | C16412 |
| R08038 | 4 2-Hydroxylamino-4,6-dinitrotoluene + Oxygen <=> 2 4,4',6,6'-Tetranitro-2,2'-azoxytoluene + 4 H2O | 4 C16393 + C00007 <=> 2 C16411 + 4 C00001 | C16393 | C16411 |
| R08050 | Ectoine + 2-Oxoglutarate + Oxygen <=> 5-Hydroxyectoine + Succinate + CO2 | C06231 + C00026 + C00007 <=> C16432 + C00042 + C00011 | C06231 | C16432 |
| R08053 | Isopentenyladenosine-5'-triphosphate + Reduced flavoprotein + Oxygen <=> trans-Zeatin riboside triphosphate + Oxidized flavoprotein + H2O | C16424 + C03024 + C00007 <=> C16428 + C03161 + C00001 | C16424 | C16428 |
| R08054 | Isopentenyladenosine-5'-diphosphate + Reduced flavoprotein + Oxygen <=> trans-Zeatin riboside diphosphate + Oxidized flavoprotein + H2O | C16426 + C03024 + C00007 <=> C16429 + C03161 + C00001 | C16426 | C16429 |
| R08055 | N6-(delta2-Isopentenyl)-adenosine 5'-monophosphate + Reduced flavoprotein + Oxygen <=> trans-Zeatin riboside monophosphate + Oxidized flavoprotein + H2O | C04713 + C03024 + C00007 <=> C16430 + C03161 + C00001 | C04713 | C16430 |
| R08066 | Isopentenyl adenosine + Reduced flavoprotein + Oxygen <=> trans-Zeatin riboside + Oxidized flavoprotein + H2O | C16427 + C03024 + C00007 <=> C16431 + C03161 + C00001 | C16427 | C16431 |
| R08068 | N6-(delta2-Isopentenyl)-adenine + Reduced flavoprotein + Oxygen <=> Zeatin + Oxidized flavoprotein + H2O | C04083 + C03024 + C00007 <=> C00371 + C03161 + C00001 | C04083 | C00371 |
| R08081 | Pinocembrin + 2-Oxoglutarate + Oxygen <=> Chrysin + Succinate + CO2 + H2O | C09827 + C00026 + C00007 <=> C10028 + C00042 + C00011 + C00001 | C09827 | C10028 |
| R08082 | Pinobanksin + 2-Oxoglutarate + Oxygen <=> Galangin + Succinate + CO2 + H2O | C09826 + C00026 + C00007 <=> C10044 + C00042 + C00011 + C00001 | C09826 | C10044 |
| R08100 | 2-Fluorobenzoate + NADH + H+ + Oxygen <=> 2-Fluorocyclohexadiene-cis,cis-1,2-diol-1-carboxylate + NAD+ | C02359 + C00004 + C00080 + C00007 <=> C16482 + C00003 | C02359 | C16482 |
| R08101 | 2-Fluorobenzoate + NADH + H+ + Oxygen <=> 6-Fluorocyclohexadiene-cis,cis-1,2-diol-1-carboxylate + NAD+ | C02359 + C00004 + C00080 + C00007 <=> C16481 + C00003 | C02359 | C16481 |
| R08103 | Fluorobenzene + NADH + Oxygen + H+ <=> 4-Fluorocyclohexadiene-cis,cis-1,2-diol + NAD+ | C11272 + C00004 + C00007 + C00080 <=> C16483 + C00003 | C11272 | C16483 |
| R08104 | Fluorobenzene + NADH + H+ + Oxygen <=> 1-Fluorocyclohexadiene-cis,cis-1,2-diol + NAD+ | C11272 + C00004 + C00080 + C00007 <=> C16484 + C00003 | C11272 | C16484 |
| R08108 | 3-Fluorobenzoate + NADH + Oxygen + H+ <=> 3-Fluorocyclohexadiene-cis,cis-1,2-diol-1-carboxylate + NAD+ | C02364 + C00004 + C00007 + C00080 <=> C16478 + C00003 | C02364 | C16478 |
| R08109 | 3-Fluorobenzoate + NADH + H+ + Oxygen <=> 5-Fluorocyclohexadiene-cis,cis-1,2-diol-1-carboxylate + NAD+ | C02364 + C00004 + C00080 + C00007 <=> C16479 + C00003 | C02364 | C16479 |
| R08110 | 4-Fluorobenzoate + NADH + H+ + Oxygen <=> 4-Fluorocyclohexadiene-cis,cis-1,2-diol-1-carboxylate + NAD+ | C02371 + C00004 + C00080 + C00007 <=> C16480 + C00003 | C02371 | C16480 |
| R08114 | 3-Fluorocatechol + Oxygen <=> 2-Fluoro-cis,cis-muconate | C16472 + C00007 <=> C16475 | C16472 | C16475 |
| R08115 | 4-Fluorocatechol + Oxygen <=> 3-Fluoro-cis,cis-muconate | C16473 + C00007 <=> C16474 | C16473 | C16474 |
| R08148 | Methyl farnesoate + Oxygen + NADPH + H+ <=> Juvenile hormone III + NADP+ + H2O | C16503 + C00007 + C00005 + C00080 <=> C09694 + C00006 + C00001 | C16503 | C09694 |
| R08160 | L-Tryptophan + 2 Oxygen + 2 NADPH + 2 H+ <=> Indole-3-acetaldehyde oxime + 3 H2O + 2 NADP+ + CO2 | C00078 + 2 C00007 + 2 C00005 + 2 C00080 <=> C02937 + 3 C00001 + 2 C00006 + C00011 | C00078 | C02937 |
| R08161 | Hexadecanoyl-[acp] + Reduced acceptor + Oxygen <=> Hexadecenoyl-[acyl-carrier protein] + Acceptor + 2 H2O | C05764 + C00030 + C00007 <=> C16520 + C00028 + 2 C00001 | C05764 | C16520 |
| R08168 | Indole-3-acetaldehyde oxime + L-Cysteine + NADPH + Oxygen + H+ <=> S-(Indolylmethylthiohydroximoyl)-L-cysteine + NADP+ + 2 H2O | C02937 + C00097 + C00005 + C00007 + C00080 <=> C16518 + C00006 + 2 C00001 | C02937 | C16518 |
| R08173 | 3'-N-Debenzoyl-2'-deoxytaxol + Reduced acceptor + Oxygen <=> 3'-N-Debenzoyltaxol + Acceptor + H2O | C16523 + C00030 + C00007 <=> C16524 + C00028 + C00001 | C16523 | C16524 |
| R08203 | Chlorophyllide + Oxygen + NADPH + H+ <=> 7-Hydroxychlorophyllide a + H2O + NADP+ | C02139 + C00007 + C00005 + C00080 <=> C16540 + C00001 + C00006 | C02139 | C16540 |
| R08204 | 7-Hydroxychlorophyllide a + Oxygen + NADPH + H+ <=> Chlorophyllide b + 2 H2O + NADP+ | C16540 + C00007 + C00005 + C00080 <=> C16541 + 2 C00001 + C00006 | C16540 | C16541 |
| R08205 | Androstenedione + NADPH + H+ + Oxygen <=> Testololactone + NADP+ + H2O | C00280 + C00005 + C00080 + C00007 <=> C04676 + C00006 + C00001 | C00280 | C04676 |
| R08206 | 17alpha-Hydroxyprogesterone + NADPH + H+ + Oxygen <=> Androstenedione + Acetate + NADP+ + H2O | C01176 + C00005 + C00080 + C00007 <=> C00280 + C00033 + C00006 + C00001 | C01176 | C00280 |
| R08207 | Marmesin + NADPH + H+ + Oxygen <=> Psoralen + NADP+ + Acetone + 2 H2O | C09276 + C00005 + C00080 + C00007 <=> C09305 + C00006 + C00207 + 2 C00001 | C09276 | C09305 |
| R08211 | Betaine aldehyde + Oxygen + H2O <=> Betaine + H2O2 | C00576 + C00007 + C00001 <=> C00719 + C00027 | C00576 | C00719 |
| R08212 | Choline + 2 Oxygen + H2O <=> Betaine + 2 H2O2 | C00114 + 2 C00007 + C00001 <=> C00719 + 2 C00027 | C00114 | C00719 |
| R08225 | Tegafur + Reduced flavoprotein + Oxygen <=> 5-FU + Succinic aldehyde + Oxidized flavoprotein + H2O | C12673 + C03024 + C00007 <=> C07649 + C16835 + C03161 + C00001 | C12673 | C07649 |
| R08235 | 6-Mercaptopurine + 2 H2O + 2 Oxygen <=> 6-Thiourate + 2 H2O2 | C02380 + 2 C00001 + 2 C00007 <=> C16613 + 2 C00027 | C02380 | C16613 |
| R08257 | Irinotecan + 2 Reduced flavoprotein + 2 Oxygen <=> NPC + Glutaral + 2 Oxidized flavoprotein + 2 H2O | C16641 + 2 C03024 + 2 C00007 <=> C16543 + C12518 + 2 C03161 + 2 C00001 | C16641 | C16543 |
| R08264 | Codeine + Reduced flavoprotein + Oxygen <=> Norcodeine + Formaldehyde + Oxidized flavoprotein + H2O | C06174 + C03024 + C00007 <=> C16576 + C00067 + C03161 + C00001 | C06174 | C16576 |
| R08265 | Morphine + Reduced flavoprotein + Oxygen <=> Normorphine + Formaldehyde + Oxidized flavoprotein + H2O | C01516 + C03024 + C00007 <=> C11785 + C00067 + C03161 + C00001 | C01516 | C11785 |
| R08266 | Tamoxifen + Oxygen + NADPH + H+ <=> Tamoxifen N-oxide + NADP+ + H2O | C07108 + C00007 + C00005 + C00080 <=> C16545 + C00006 + C00001 | C07108 | C16545 |
| R08267 | Tamoxifen + NADPH + Oxygen + H+ <=> Hydroxytamoxifen + NADP+ + H2O | C07108 + C00005 + C00007 + C00080 <=> C05011 + C00006 + C00001 | C07108 | C05011 |
| R08270 | N-Desmethyltamoxifen + NADPH + Oxygen + H+ <=> 4-Hydroxy-N-desmethyltamoxifen + NADP+ + H2O | C16546 + C00005 + C00007 + C00080 <=> C16547 + C00006 + C00001 | C16546 | C16547 |
| R08271 | Tamoxifen + NADPH + Oxygen + H+ <=> alpha-Hydroxytamoxifen + NADP+ + H2O | C07108 + C00005 + C00007 + C00080 <=> C16544 + C00006 + C00001 | C07108 | C16544 |
| R08274 | N-Desmethyltamoxifen + NADPH + H+ + Oxygen <=> alpha-Hydroxy-N-desmethyltamoxifen + H2O + NADP+ | C16546 + C00005 + C00080 + C00007 <=> C16549 + C00001 + C00006 | C16546 | C16549 |
| R08275 | Cyclophosphamide + NADPH + H+ + Oxygen <=> 4-Hydroxycyclophosphamide + NADP+ + H2O | C07888 + C00005 + C00080 + C00007 <=> C07643 + C00006 + C00001 | C07888 | C07643 |
| R08285 | Ifosfamide + NADPH + H+ + Oxygen <=> 4-Hydroxyifosfamide + NADP+ + H2O | C07047 + C00005 + C00080 + C00007 <=> C16553 + C00006 + C00001 | C07047 | C16553 |
| R08294 | Lidocaine + NADPH + Oxygen + H+ <=> 3-Hydroxylidocaine + NADP+ + H2O | C07073 + C00005 + C00007 + C00080 <=> C16560 + C00006 + C00001 | C07073 | C16560 |
| R08303 | Felbamate + NADPH + H+ + Oxygen <=> p-Hydroxyfelbamate + NADP+ + H2O | C07501 + C00005 + C00080 + C00007 <=> C16584 + C00006 + C00001 | C07501 | C16584 |
| R08304 | Felbamate + NADPH + H+ + Oxygen <=> 2-Hydroxyfelbamate + NADP+ + H2O | C07501 + C00005 + C00080 + C00007 <=> C16582 + C00006 + C00001 | C07501 | C16582 |
| R08312 | Carbamazepine + NADPH + H+ + Oxygen <=> Carbamazepine-10,11-epoxide + NADP+ + H2O | C06868 + C00005 + C00080 + C00007 <=> C07496 + C00006 + C00001 | C06868 | C07496 |
| R08313 | Carbamazepine + NADPH + H+ + Oxygen <=> 3-Hydroxycarbamazepine + NADP+ + H2O | C06868 + C00005 + C00080 + C00007 <=> C16602 + C00006 + C00001 | C06868 | C16602 |
| R08325 | Valproic acid + NADPH + H+ + Oxygen <=> 4-Hydroxyvalproic acid + NADP+ + H2O | C07185 + C00005 + C00080 + C00007 <=> C16649 + C00006 + C00001 | C07185 | C16649 |
| R08326 | Valproic acid + NADPH + H+ + Oxygen <=> 5-Hydroxyvalproic acid + NADP+ + H2O | C07185 + C00005 + C00080 + C00007 <=> C16650 + C00006 + C00001 | C07185 | C16650 |
| R08327 | Valproic acid + NADPH + H+ + Oxygen <=> 3-Hydroxyvalproic acid + NADP+ + H2O | C07185 + C00005 + C00080 + C00007 <=> C16651 + C00006 + C00001 | C07185 | C16651 |
| R08346 | Citalopram + Oxygen + H2O <=> Citalopram aldehyde + Dimethylamine + H2O2 | C07572 + C00007 + C00001 <=> C16612 + C00543 + C00027 | C07572 | C16612 |
| R08347 | Demethylcitalopram + Oxygen + H2O <=> Citalopram aldehyde + Methylamine + H2O2 | C16608 + C00007 + C00001 <=> C16612 + C00218 + C00027 | C16608 | C16612 |
| R08348 | Didemethylcitalopram + H2O + Oxygen <=> Citalopram aldehyde + NH3 + H2O2 | C16609 + C00001 + C00007 <=> C16612 + C00014 + C00027 | C16609 | C16612 |
| R08349 | Citalopram aldehyde + H2O + Oxygen <=> Citalopram propionic acid + H2O2 | C16612 + C00001 + C00007 <=> C16610 + C00027 | C16612 | C16610 |
| R08384 | 9-cis-Retinal + Oxygen + H2O <=> 9-cis-Retinoic acid + H2O2 | C16681 + C00007 + C00001 <=> C15493 + C00027 | C16681 | C15493 |
| R08390 | Retinoate + NADPH + H+ + Oxygen <=> all-trans-18-Hydroxyretinoic acid + NADP+ + H2O | C00777 + C00005 + C00080 + C00007 <=> C16679 + C00006 + C00001 | C00777 | C16679 |
| R08391 | Retinoate + NADPH + H+ + Oxygen <=> all-trans-5,6-Epoxyretinoic acid + NADP+ + H2O | C00777 + C00005 + C00080 + C00007 <=> C16680 + C00006 + C00001 | C00777 | C16680 |
| R08392 | Retinoate + NADPH + H+ + Oxygen <=> all-trans-4-Hydroxyretinoic acid + NADP+ + H2O | C00777 + C00005 + C00080 + C00007 <=> C16677 + C00006 + C00001 | C00777 | C16677 |
| R08408 | 1-Methylnicotinamide + Oxygen + H2O <=> N1-Methyl-2-pyridone-5-carboxamide + H2O2 + H+ | C02918 + C00007 + C00001 <=> C05842 + C00027 + C00080 | C02918 | C05842 |
| R08505 | Cholesterol + Oxygen + NADPH + H+ <=> Cholest-5-ene-3beta,26-diol + NADP+ + H2O | C00187 + C00007 + C00005 + C00080 <=> C15610 + C00006 + C00001 | C00187 | C15610 |
| R08517 | 17alpha-Hydroxypregnenolone + Reduced acceptor + Oxygen <=> Dehydroepiandrosterone + Acetate + Acceptor + H2O | C05138 + C00030 + C00007 <=> C01227 + C00033 + C00028 + C00001 | C05138 | C01227 |
| R08518 | 17alpha-Hydroxyprogesterone + Reduced acceptor + Oxygen <=> Androstenedione + Acetate + Acceptor + H2O | C01176 + C00030 + C00007 <=> C00280 + C00033 + C00028 + C00001 | C01176 | C00280 |
| R08527 | (5Z,8Z,11Z,14Z)-Icosatetraenoic acid + Oxygen <=> Leukotriene A4 + H2O | C00219 + C00007 <=> C00909 + C00001 | C00219 | C00909 |
| R08604 | Tryptamine + Oxygen + NADPH <=> N-Hydroxyl-tryptamine + H2O + NADP+ | C00398 + C00007 + C00005 <=> C17203 + C00001 + C00006 | C00398 | C17203 |
| R08607 | Indole-3-acetaldehyde oxime + NADPH + Oxygen + H+ <=> Indole-3-acetaldoxime N-oxide + NADP+ + H2O | C02937 + C00005 + C00007 + C00080 <=> C17204 + C00006 + C00001 | C02937 | C17204 |
| R08610 | 5-Hydroxymethyluracil + 2-Oxoglutarate + Oxygen <=> Uracil 5-carbaldehyde + Succinate + CO2 + H2O | C03088 + C00026 + C00007 <=> C17206 + C00042 + C00011 + C00001 | C03088 | C17206 |
| R08611 | Uracil 5-carbaldehyde + 2-Oxoglutarate + Oxygen <=> Uracil 5-carboxylate + Succinate + CO2 | C17206 + C00026 + C00007 <=> C03030 + C00042 + C00011 | C17206 | C03030 |
| R08652 | L-Phenylalanine + 2 Oxygen + 2 NADPH + 2 H+ <=> (Z)-Phenylacetaldehyde oxime + 3 H2O + 2 NADP+ + CO2 | C00079 + 2 C00007 + 2 C00005 + 2 C00080 <=> C16075 + 3 C00001 + 2 C00006 + C00011 | C00079 | C16075 |
| R08653 | (Z)-Phenylacetaldehyde oxime + L-Cysteine + NADPH + Oxygen + H+ <=> S-(Phenylacetothiohydroximoyl)-L-cysteine + NADP+ + 2 H2O | C16075 + C00097 + C00005 + C00007 + C00080 <=> C17237 + C00006 + 2 C00001 | C16075 | C17237 |
| R08655 | L-Homophenylalanine + 2 Oxygen + 2 NADPH + 2 H+ <=> 3-Phenylpropionaldoxim + 2 NADP+ + CO2 + 3 H2O | C17235 + 2 C00007 + 2 C00005 + 2 C00080 <=> C17236 + 2 C00006 + C00011 + 3 C00001 | C17235 | C17236 |
| R08656 | L-Tyrosine + 2 Oxygen + 2 NADPH + 2 H+ <=> (Z)-4-Hydroxyphenylacetaldehyde-oxime + 2 NADP+ + 3 H2O + CO2 | C00082 + 2 C00007 + 2 C00005 + 2 C00080 <=> C04353 + 2 C00006 + 3 C00001 + C00011 | C00082 | C04353 |
| R08659 | (Z)-4-Hydroxyphenylacetaldehyde-oxime + Oxygen + NADPH + L-Cysteine + H+ <=> S-(Hydroxyphenylacetothiohydroximoyl)-L-cysteine + NADP+ + 2 H2O | C04353 + C00007 + C00005 + C00097 + C00080 <=> C17238 + C00006 + 2 C00001 | C04353 | C17238 |
| R08663 | L-Valine + 2 Oxygen + 2 NADPH + 2 H+ <=> 2-Methylpropanal oxime + 3 H2O + 2 NADP+ + CO2 | C00183 + 2 C00007 + 2 C00005 + 2 C00080 <=> C03219 + 3 C00001 + 2 C00006 + C00011 | C00183 | C03219 |
| R08664 | L-Leucine + 2 Oxygen + 2 NADPH + 2 H+ <=> 3-Methylbutyraldehyde oxime + 3 H2O + 2 NADP+ + CO2 | C00123 + 2 C00007 + 2 C00005 + 2 C00080 <=> C17255 + 3 C00001 + 2 C00006 + C00011 | C00123 | C17255 |
| R08665 | Homomethionine + 2 Oxygen + 2 NADPH + 2 H+ <=> 4-Methylthiobutanaldoxime + 3 H2O + 2 NADP+ + CO2 | C17213 + 2 C00007 + 2 C00005 + 2 C00080 <=> C17241 + 3 C00001 + 2 C00006 + C00011 | C17213 | C17241 |
| R08666 | 4-Methylthiobutanaldoxime + L-Cysteine + NADPH + Oxygen + H+ <=> S-(4-Methylthiobutylthiohydroximoyl)-L-cysteine + NADP+ + 2 H2O | C17241 + C00097 + C00005 + C00007 + C00080 <=> C17242 + C00006 + 2 C00001 | C17241 | C17242 |
| R08670 | Dihomomethionine + 2 Oxygen + 2 NADPH + 2 H+ <=> 5-Methylthiopentanaldoxime + 3 H2O + 2 NADP+ + CO2 | C17217 + 2 C00007 + 2 C00005 + 2 C00080 <=> C17245 + 3 C00001 + 2 C00006 + C00011 | C17217 | C17245 |
| R08672 | Trihomomethionine + 2 Oxygen + 2 NADPH + 2 H+ <=> 6-Methylthiohexanaldoxime + 3 H2O + 2 NADP+ + CO2 | C17221 + 2 C00007 + 2 C00005 + 2 C00080 <=> C17246 + 3 C00001 + 2 C00006 + C00011 | C17221 | C17246 |
| R08673 | Tetrahomomethionine + 2 Oxygen + 2 NADPH + 2 H+ <=> 7-Methylthioheptanaldoxime + 3 H2O + 2 NADP+ + CO2 | C17225 + 2 C00007 + 2 C00005 + 2 C00080 <=> C17249 + 3 C00001 + 2 C00006 + C00011 | C17225 | C17249 |
| R08674 | Pentahomomethionine + 2 Oxygen + 2 NADPH + 2 H+ <=> 8-Methylthiooctanaldoxime + 3 H2O + 2 NADP+ + CO2 | C17229 + 2 C00007 + 2 C00005 + 2 C00080 <=> C17251 + 3 C00001 + 2 C00006 + C00011 | C17229 | C17251 |
| R08675 | Hexahomomethionine + 2 Oxygen + 2 NADPH + 2 H+ <=> 9-Methylthiononanaldoxime + 3 H2O + 2 NADP+ + CO2 | C17233 + 2 C00007 + 2 C00005 + 2 C00080 <=> C17253 + 3 C00001 + 2 C00006 + C00011 | C17233 | C17253 |
| R08678 | S-Sulfanylglutathione + Oxygen + H2O <=> Glutathione + Sulfite | C17267 + C00007 + C00001 <=> C00051 + C00094 | C17267 | C00051 |
| R08684 | Amino acid + 2 Oxygen + 2 NADPH + 2 H+ <=> Aldoxime + 2 NADP+ + 3 H2O + CO2 | C00045 + 2 C00007 + 2 C00005 + 2 C00080 <=> C02658 + 2 C00006 + 3 C00001 + C00011 | C00045 | C02658 |
| R08685 | Aldoxime + NADPH + Oxygen + L-Cysteine + H+ <=> S-Alkyl-thiohydroximate + NADP+ + 2 H2O | C02658 + C00005 + C00007 + C00097 + C00080 <=> C17262 + C00006 + 2 C00001 | C02658 | C17262 |
| R08702 | tRNA containing 5-carboxymethylaminomethyl-2-thiouridine + Oxygen + H2O <=> tRNA containing 5-aminomethyl-2-thiouridine + Glyoxylate + H2O2 | C17323 + C00007 + C00001 <=> C11478 + C00048 + C00027 | C17323 | C11478 |
| R08725 | 7alpha,26-Dihydroxy-4-cholesten-3-one + 2 Oxygen + 2 NADPH + 2 H+ <=> 7alpha-Hydroxy-3-oxo-4-cholestenoate + 2 NADP+ + 3 H2O | C17336 + 2 C00007 + 2 C00005 + 2 C00080 <=> C17337 + 2 C00006 + 3 C00001 | C17336 | C17337 |
| R08726 | Cholest-5-ene-3beta,26-diol + 2 Oxygen + 2 NADPH + 2 H+ <=> 3beta-Hydroxy-5-cholestenoate + 2 NADP+ + 3 H2O | C15610 + 2 C00007 + 2 C00005 + 2 C00080 <=> C17333 + 2 C00006 + 3 C00001 | C15610 | C17333 |
| R08727 | 3beta-Hydroxy-5-cholestenoate + Oxygen + NADPH + H+ <=> 3beta,7alpha-Dihydroxy-5-cholestenoate + NADP+ + H2O | C17333 + C00007 + C00005 + C00080 <=> C17335 + C00006 + C00001 | C17333 | C17335 |
| R08760 | 3alpha,7alpha-Dihydroxy-5beta-cholestan-26-al + NADPH + Oxygen + H+ <=> 3alpha,7alpha-Dihydroxy-5beta-cholestanate + NADP+ + H2O | C05445 + C00005 + C00007 + C00080 <=> C04554 + C00006 + C00001 | C05445 | C04554 |
| R08761 | 3alpha,7alpha,12alpha-Trihydroxy-5beta-cholestan-26-al + NADPH + Oxygen + H+ <=> 3alpha,7alpha,12alpha-Trihydroxy-5beta-cholestanoate + NADP+ + H2O | C01301 + C00005 + C00007 + C00080 <=> C04722 + C00006 + C00001 | C01301 | C04722 |
| R08762 | 2 2-Aminophenol + Oxygen <=> 2 6-Iminocyclohexa-2,4-dienone + 2 H2O | 2 C01987 + C00007 <=> 2 C17500 + 2 C00001 | C01987 | C17500 |
| R08764 | 6-Hydroxynicotinate + NADH + Oxygen + H+ <=> 2,5-Dihydroxypyridine + NAD+ + CO2 + H2O | C01020 + C00004 + C00007 + C00080 <=> C01059 + C00003 + C00011 + C00001 | C01020 | C01059 |
| R08768 | 2-Polyprenylphenol + Oxygen + NADPH <=> 2-Polyprenyl-6-hydroxyphenol + NADP+ + H2O | C05807 + C00007 + C00005 <=> C17551 + C00006 + C00001 | C05807 | C17551 |
| R08770 | 4-Hydroxy-3-polyprenylbenzoate + Oxygen <=> 3-Polyprenyl-4,5-dihydroxybenzoate | C05848 + C00007 <=> C17554 | C05848 | C17554 |
| R08773 | 2-Polyprenyl-6-methoxyphenol + Oxygen <=> 2-Polyprenyl-6-methoxy-1,4-benzoquinone + H2O | C17552 + C00007 <=> C17560 + C00001 | C17552 | C17560 |
| R08775 | 2-Polyprenyl-3-methyl-6-methoxy-1,4-benzoquinone + Oxygen + NADPH + H+ <=> 2-Polyprenyl-3-methyl-5-hydroxy-6-methoxy-1,4-benzoquinone + NADP+ + H2O | C17561 + C00007 + C00005 + C00080 <=> C17562 + C00006 + C00001 | C17561 | C17562 |
| R08785 | Geraniol + Reduced flavoprotein + Oxygen <=> 10-Hydroxygeraniol + Oxidized flavoprotein + H2O | C01500 + C03024 + C00007 <=> C17621 + C03161 + C00001 | C01500 | C17621 |
| R08790 | (S)-Reticuline + NADPH + Oxygen + H+ <=> (S)-Corytuberine + NADP+ + 2 H2O | C02105 + C00005 + C00007 + C00080 <=> C17591 + C00006 + 2 C00001 | C02105 | C17591 |
| R08795 | Tetrahydropalmatine + 2 Oxygen + H+ <=> Palmatine + 2 H2O2 | C02890 + 2 C00007 + C00080 <=> C05315 + 2 C00027 | C02890 | C05315 |
| R08815 | Cinnamoyl-CoA + Oxygen + NADPH + H+ <=> p-Coumaroyl-CoA + NADP+ + H2O | C00540 + C00007 + C00005 + C00080 <=> C00223 + C00006 + C00001 | C00540 | C00223 |
| R08826 | Portulacaxanthin II + Oxygen + Dopaxanthin <=> Dopaxanthin + H2O + Dopaxanthin quinone | C08565 + C00007 + C08543 <=> C08543 + C00001 + C17753 | C08565 | C08543 |
| R08828 | 2 Dopaxanthin + Oxygen <=> 2 Dopaxanthin quinone + 2 H2O | 2 C08543 + C00007 <=> 2 C17753 + 2 C00001 | C08543 | C17753 |
| R08832 | 2 Dopamine + Oxygen <=> 2 Dopamine quinone + 2 H2O | 2 C03758 + C00007 <=> 2 C17755 + 2 C00001 | C03758 | C17755 |
| R08836 | 3,4-Dihydroxy-L-phenylalanine + Oxygen <=> 4,5-seco-Dopa | C00355 + C00007 <=> C17758 | C00355 | C17758 |
| R08838 | Benzoate + NADPH + Oxygen <=> Salicylate + NADP+ + H2O | C00180 + C00005 + C00007 <=> C00805 + C00006 + C00001 | C00180 | C00805 |
| R08840 | Teasterone + Reduced flavoprotein + Oxygen <=> 7-Oxateasterone + Oxidized flavoprotein + H2O | C15791 + C03024 + C00007 <=> C17733 + C03161 + C00001 | C15791 | C17733 |
| R08841 | Typhasterol + Reduced flavoprotein + Oxygen <=> 7-Oxatyphasterol + Oxidized flavoprotein + H2O | C15793 + C03024 + C00007 <=> C17735 + C03161 + C00001 | C15793 | C17735 |
| R08851 | (+)-epi-Isozizaene + NADPH + H+ + Oxygen <=> (5S)-Albaflavenol + NADP+ + H2O | C16269 + C00005 + C00080 + C00007 <=> C17953 + C00006 + C00001 | C16269 | C17953 |
| R08852 | (5S)-Albaflavenol + NADPH + H+ + Oxygen <=> Albaflavenone + NADP+ + 2 H2O | C17953 + C00005 + C00080 + C00007 <=> C17954 + C00006 + 2 C00001 | C17953 | C17954 |
| R08853 | (+)-epi-Isozizaene + NADPH + H+ + Oxygen <=> (5R)-Albaflavenol + NADP+ + H2O | C16269 + C00005 + C00080 + C00007 <=> C17955 + C00006 + C00001 | C16269 | C17955 |
| R08854 | (5R)-Albaflavenol + NADPH + H+ + Oxygen <=> Albaflavenone + 2 H2O + NADP+ | C17955 + C00005 + C00080 + C00007 <=> C17954 + 2 C00001 + C00006 | C17955 | C17954 |
| R08889 | 8'-apo-beta-Carotenol + Oxygen <=> 14'-apo-beta-Carotenal + 7-Hydroxy-6-methylhepta-3,5-dienal | C06733 + C00007 <=> C06734 + C18011 | C06733 | C06734 |
| R08896 | Paromamine + Oxygen <=> 6'-Dehydro-6'-oxoparomamine + H2O2 | C01743 + C00007 <=> C17583 + C00027 | C01743 | C17583 |
| R08902 | 6'''-Deamino-6'''-hydroxyneomycin C + Oxygen <=> 6'''-Deamino-6'''-dehydro-6'''-oxoneomycin C + H2O2 | C17588 + C00007 <=> C17589 + C00027 | C17588 | C17589 |
| R08908 | gamma-L-Glutamyl-4-aminobutyryl-[acp] + Reduced FMN + Oxygen <=> gamma-L-Glutamyl-4-amino-2-hydroxybutyryl-[acp] + FMN + H2O | C18008 + C01847 + C00007 <=> C18009 + C00061 + C00001 | C18008 | C18009 |
| R08912 | 2 Flaviolin + NADPH + H+ + Oxygen <=> 3,3'-Biflaviolin + NADP+ + 2 H2O | 2 C18012 + C00005 + C00080 + C00007 <=> C18013 + C00006 + 2 C00001 | C18012 | C18013 |
| R08913 | 2 Flaviolin + NADPH + H+ + Oxygen <=> 3,8'-Biflaviolin + NADP+ + 2 H2O | 2 C18012 + C00005 + C00080 + C00007 <=> C18014 + C00006 + 2 C00001 | C18012 | C18014 |
| R08920 | 2 3,4-Dihydroxy-L-phenylalanine + Oxygen <=> 2 3-(3,4-Dihydroxyphenyl)pyruvate + 2 NH3 | 2 C00355 + C00007 <=> 2 C04045 + 2 C00014 | C00355 | C04045 |
| R08921 | Pheophorbide a + NADPH + H+ + Oxygen <=> Red chlorophyll catabolite + NADP+ | C18021 + C00005 + C00080 + C00007 <=> C18022 + C00006 | C18021 | C18022 |
| R08922 | Sophoraflavanone B + NADPH + H+ + Oxygen <=> Leachianone G + NADP+ + H2O | C18023 + C00005 + C00080 + C00007 <=> C18024 + C00006 + C00001 | C18023 | C18024 |
| R08923 | Pulegone + NADPH + H+ + Oxygen <=> (+)-Menthofuran + NADP+ + 2 H2O | C09893 + C00005 + C00080 + C00007 <=> C18025 + C00006 + 2 C00001 | C09893 | C18025 |
| R08925 | (-)-Menthone + NADPH + H+ + Oxygen <=> (4R,7S)-7-Isopropyl-4-methyloxepan-2-one + NADP+ + H2O | C00843 + C00005 + C00080 + C00007 <=> C18027 + C00006 + C00001 | C00843 | C18027 |
| R08943 | Pregnenolone + NADPH + H+ + Oxygen <=> 7alpha-Hydroxypregnenolone + NADP+ + H2O | C01953 + C00005 + C00080 + C00007 <=> C18038 + C00006 + C00001 | C01953 | C18038 |
| R08949 | 11-Deoxycorticosterone + 3 Reduced adrenal ferredoxin + 3 Oxygen <=> Aldosterone + 3 Oxidized adrenal ferredoxin + 4 H2O | C03205 + 3 C00662 + 3 C00007 <=> C01780 + 3 C00667 + 4 C00001 | C03205 | C01780 |
| R08961 | Dehydroepiandrosterone + Oxygen + NADPH + H+ <=> 7alpha-Hydroxydehydroepiandrosterone + NADP+ + H2O | C01227 + C00007 + C00005 + C00080 <=> C18045 + C00006 + C00001 | C01227 | C18045 |
| R08965 | 2 5,6-Dihydroxyindole-2-carboxylate + Oxygen <=> 2 5,6-Indolequinone-2-carboxylic acid + 2 H2O | 2 C04185 + C00007 <=> 2 C17938 + 2 C00001 | C04185 | C17938 |
| R08972 | DIBOA-glucoside + 2-Oxoglutarate + Oxygen <=> TRIBOA-glucoside + Succinate + CO2 | C15772 + C00026 + C00007 <=> C18062 + C00042 + C00011 | C15772 | C18062 |
| R09042 | Geranylhydroquinone + NADPH + Oxygen + H+ <=> 3''-Hydroxy-geranylhydroquinone + H2O + NADP+ | C10793 + C00005 + C00007 + C00080 <=> C18132 + C00001 + C00006 | C10793 | C18132 |
| R09074 | N1-Acetylspermidine + Oxygen + H2O <=> Putrescine + 3-Acetamidopropanal + H2O2 | C00612 + C00007 + C00001 <=> C00134 + C18170 + C00027 | C00612 | C00134 |
| R09075 | N8-Acetylspermidine + Oxygen + H2O <=> 1,3-Diaminopropane + N4-Acetylaminobutanal + H2O2 | C01029 + C00007 + C00001 <=> C00986 + C05936 + C00027 | C01029 | C00986 |
| R09076 | Spermine + Oxygen + H2O <=> Spermidine + 3-Aminopropanal + H2O2 | C00750 + C00007 + C00001 <=> C00315 + C05665 + C00027 | C00750 | C00315 |
| R09077 | Spermidine + Oxygen + H2O <=> Putrescine + 3-Aminopropanal + H2O2 | C00315 + C00007 + C00001 <=> C00134 + C05665 + C00027 | C00315 | C00134 |
| R09083 | Reduced FMN + Oxygen <=> Dimethylbenzimidazole + D-Erythrose 4-phosphate + H2O | C01847 + C00007 <=> C03114 + C00279 + C00001 | C01847 | C03114 |
| R09122 | Geranylgeraniol + NADPH + H+ + Oxygen <=> 18-Hydroxygeranylgeraniol + NADP+ + H2O | C09094 + C00005 + C00080 + C00007 <=> C13273 + C00006 + C00001 | C09094 | C13273 |
| R09125 | 2,5-Dihydroxypyridine + Oxygen <=> N-formylmaleamic acid | C01059 + C00007 <=> C18232 | C01059 | C18232 |
| R09130 | 1,2,3,4-Tetrachlorobenzene + Oxygen + NADH + H+ <=> cis-Chlorobenzene dihydrodiol + NAD+ | C18236 + C00007 + C00004 + C00080 <=> C18238 + C00003 | C18236 | C18238 |
| R09131 | 1,2,3,4-Tetrachlorobenzene + Oxygen + NADPH + H+ <=> cis-Chlorobenzene dihydrodiol + NADP+ | C18236 + C00007 + C00005 + C00080 <=> C18238 + C00006 | C18236 | C18238 |
| R09134 | Tetrachlorocatechol + Oxygen <=> Tetrachloro-cis,cis-muconate | C18240 + C00007 <=> C18241 | C18240 | C18241 |
| R09141 | Trichloroethene + NADH + H+ + Oxygen <=> TCE epoxide + NAD+ + H2O | C06790 + C00004 + C00080 + C00007 <=> C11148 + C00003 + C00001 | C06790 | C11148 |
| R09148 | 1,1,1-Trichloroethane + NADH + H+ + Oxygen <=> Trichloroethanol + NAD+ + H2O | C18246 + C00004 + C00080 + C00007 <=> C07490 + C00003 + C00001 | C18246 | C07490 |
| R09159 | Benzo[a]pyrene + Oxygen + NADH + H+ <=> Benzo[a]pyrene-cis-9,10-dihydrodiol + NAD+ | C07535 + C00007 + C00004 + C00080 <=> C18276 + C00003 | C07535 | C18276 |
| R09160 | Benzo[a]pyrene + Oxygen + NADH + H+ <=> Benzo[a]pyrene-cis-7,8-dihydrodiol + NAD+ | C07535 + C00007 + C00004 + C00080 <=> C18275 + C00003 | C07535 | C18275 |
| R09161 | Pyrene + Oxygen <=> cis-4,5-Dihydroxy-4,5-dihydropyrene | C14335 + C00007 <=> C18249 | C14335 | C18249 |
| R09163 | 4,5-Dihydroxypyrene + Oxygen <=> Phenanthrene-4,5-dicarboxylate | C18250 + C00007 <=> C18252 | C18250 | C18252 |
| R09165 | Phenanthrene-4-carboxylate + Oxygen + NADH + H+ <=> cis-3,4-Phenanthrenedihydrodiol-4-carboxylate + NAD+ | C18253 + C00007 + C00004 + C00080 <=> C18256 + C00003 | C18253 | C18256 |
| R09167 | Pyrene + Oxygen <=> Pyrene-4,5-oxide + H2O | C14335 + C00007 <=> C18254 + C00001 | C14335 | C18254 |
| R09169 | Pyrene + Oxygen <=> Pyrene-1,2-oxide + H2O | C14335 + C00007 <=> C18267 + C00001 | C14335 | C18267 |
| R09171 | 1-Hydroxypyrene + Oxygen <=> 1-Hydroxypyrene-6,7-oxide + H2O | C14519 + C00007 <=> C18265 + C00001 | C14519 | C18265 |
| R09174 | 1-Hydroxypyrene + Oxygen <=> 1-Hydroxypyrene-7,8-oxide + H2O | C14519 + C00007 <=> C18266 + C00001 | C14519 | C18266 |
| R09178 | 1-Methoxypyrene + Oxygen <=> 1-Methoxypyrene-6,7-oxide + H2O | C18259 + C00007 <=> C18262 + C00001 | C18259 | C18262 |
| R09182 | Benzo[a]pyrene-cis-7,8-dihydrodiol + Oxygen <=> cis-4-(7-Hydroxtpyren-8-yl)-2-oxobut-3-enoate | C18275 + C00007 <=> C18271 | C18275 | C18271 |
| R09184 | 9,10-Dihydroxybenzo[a]pyrene + Oxygen <=> cis-4-(8-Hydroxypyren-7-yl)-2-oxobut-3-enoate | C18278 + C00007 <=> C18272 | C18278 | C18272 |
| R09190 | 6-Methylpretetramide + 2 NADPH + 2 H+ + 2 Oxygen <=> 4-Keto-anhydrotetracycline + 2 NADP+ + 2 H2O | C06629 + 2 C00005 + 2 C00080 + 2 C00007 <=> C06627 + 2 C00006 + 2 C00001 | C06629 | C06627 |
| R09193 | Anhydrochlortetracycline + Oxygen + NADPH + H+ <=> 5a,11a-Dehydrochlortetracycline + NADP+ + H2O | C18297 + C00007 + C00005 + C00080 <=> C18298 + C00006 + C00001 | C18297 | C18298 |
| R09201 | 2,4-Dichlorotoluene + NADH + Oxygen + H+ <=> 4,6-Dichloro-3-methyl-cis-1,2-dihydroxycyclohexa-3,5-diene + NAD+ | C18300 + C00004 + C00007 + C00080 <=> C18301 + C00003 | C18300 | C18301 |
| R09204 | Benzo[a]pyrene + Oxygen + NADH + H+ <=> Benzo[a]pyrene-cis-4,5-dihydrodiol + NAD+ | C07535 + C00007 + C00004 + C00080 <=> C18270 + C00003 | C07535 | C18270 |
| R09206 | 4,5-Dihydroxybenzo[a]pyrene + Oxygen <=> 4,5-Chrysenedicarboxylate | C18279 + C00007 <=> C18277 | C18279 | C18277 |
| R09209 | Benzo[a]pyrene + NADH + H+ + Oxygen <=> Benzo[a]pyrene-cis-11,12-dihydrodiol + NAD+ | C07535 + C00004 + C00080 + C00007 <=> C18284 + C00003 | C07535 | C18284 |
| R09210 | Benzo[a]pyrene + Oxygen <=> Benzo[a]pyrene-11,12-epoxide | C07535 + C00007 <=> C18286 | C07535 | C18286 |
| R09214 | 4,6-Dichloro-3-methylcatechol + Oxygen <=> 3,5-Dichloro-3-methylmuconate | C18302 + C00007 <=> C18303 | C18302 | C18303 |
| R09227 | Phthalate + Oxygen + NADH + H+ <=> Phthalate 3,4-cis-dihydrodiol + NAD+ | C01606 + C00007 + C00004 + C00080 <=> C18314 + C00003 | C01606 | C18314 |
| R09233 | 2,4-Dinitrotoluene + NADH + Oxygen + H+ <=> 4-Methyl-5-nitrocatechol + NAD+ + Nitrite | C11006 + C00004 + C00007 + C00080 <=> C18315 + C00003 + C00088 | C11006 | C18315 |
| R09234 | 2 4-Methyl-5-nitrocatechol + 2 NADP+ + Oxygen + 2 H+ <=> 2 2-Hydroxy-5-methylquinone + 2 NADPH + 2 Nitrite | 2 C18315 + 2 C00006 + C00007 + 2 C00080 <=> 2 C18316 + 2 C00005 + 2 C00088 | C18315 | C18316 |
| R09237 | 2,4,5-Trihydroxytoluene + Oxygen <=> cis,cis-2,4-Dihydroxy-5-methyl-6-oxo-2,4-hexadienoate | C18317 + C00007 <=> C18318 | C18317 | C18318 |
| R09272 | 3-O-Methylgallate + Oxygen <=> 4-Carboxy-2-hydroxy-6-methoxy-6-oxohexa-2,4-dienoate | C05616 + C00007 <=> C18345 | C05616 | C18345 |
| R09275 | 5,5'-Dehydrodivanillate + Oxygen + NADPH + H+ <=> 2,2',3-Trihydroxy-3'-methoxy-5,5'-dicarboxybiphenyl + NADP+ + H2O + Formaldehyde | C18347 + C00007 + C00005 + C00080 <=> C18348 + C00006 + C00001 + C00067 | C18347 | C18348 |
| R09276 | 2,2',3-Trihydroxy-3'-methoxy-5,5'-dicarboxybiphenyl + Oxygen <=> 4-[2-(5-Carboxy-2-hydroxy-3-methoxyphenyl)-2-oxoethylidene]-2-hydroxy-2-pentenedioate | C18348 + C00007 <=> C18349 | C18348 | C18349 |
| R09313 | Dihydrokalafungin dihydroquinone form + Reduced FMN + Oxygen <=> DHK-OH + FMN + H2O | C18360 + C01847 + C00007 <=> C18358 + C00061 + C00001 | C18360 | C18358 |
| R09314 | 6-Deoxydihydrokalafungin + Reduced FMN + Oxygen <=> Dihydrokalafungin + FMN + H2O | C12435 + C01847 + C00007 <=> C12436 + C00061 + C00001 | C12435 | C12436 |
| R09327 | 12-Deoxynogalonic acid + Oxygen <=> Nogalonic acid + H2O | C18629 + C00007 <=> C12416 + C00001 | C18629 | C12416 |
| R09334 | Aclacinomycin N + Oxygen <=> Aclacinomycin A + Hydrogen peroxide | C18637 + C00007 <=> C18638 + C00027 | C18637 | C18638 |
| R09335 | Aclacinomycin A + Oxygen <=> Aclacinomycin Y + Hydrogen peroxide | C18638 + C00007 <=> C18639 + C00027 | C18638 | C18639 |
| R09338 | 15-Demethoxy-epsilon-rhodomycin + Oxygen + 2 Glutathione <=> beta-Rhodomycin + CO2 + H2O + Glutathione disulfide | C18641 + C00007 + 2 C00051 <=> C18642 + C00011 + C00001 + C00127 | C18641 | C18642 |
| R09353 | Premithramycin B + NADPH + H+ + Oxygen <=> Mithramycin DK + NADP+ + CO2 | C12388 + C00005 + C00080 + C00007 <=> C18711 + C00006 + C00011 | C12388 | C18711 |
| R09363 | L-Arginine + 2-Oxoglutarate + Oxygen <=> beta-Hydroxyarginine + Succinate + CO2 | C00062 + C00026 + C00007 <=> C18473 + C00042 + C00011 | C00062 | C18473 |
| R09385 | (-)-Limonene + NADH + H+ + Oxygen <=> (4S)-Limonene-1,2-epoxide + NAD+ + H2O | C00521 + C00004 + C00080 + C00007 <=> C19081 + C00003 + C00001 | C00521 | C19081 |
| R09389 | (-)-Limonene + NADPH + Oxygen + H+ <=> (4S)-Limonene-1,2-epoxide + NADP+ + H2O | C00521 + C00005 + C00007 + C00080 <=> C19081 + C00006 + C00001 | C00521 | C19081 |
| R09390 | (1R,4S)-1-Hydroxy-2-oxolimonene + NADPH + Oxygen + H+ <=> (4S)-7-Hydroxy-4-isopropenyl-7-methyl-2-oxo-oxepanone + NADP+ + H2O | C19083 + C00005 + C00007 + C00080 <=> C19084 + C00006 + C00001 | C19083 | C19084 |
| R09393 | (+)-Limonene + NADPH + Oxygen + H+ <=> Limonene-1,2-epoxide + NADP+ + H2O | C06099 + C00005 + C00007 + C00080 <=> C07271 + C00006 + C00001 | C06099 | C07271 |
| R09402 | Codeine + 2-Oxoglutarate + Oxygen <=> Morphine + Formaldehyde + Succinate + CO2 | C06174 + C00026 + C00007 <=> C01516 + C00067 + C00042 + C00011 | C06174 | C01516 |
| R09403 | L-Isoleucine + 2 Oxygen + 2 NADPH + 2 H+ <=> 2-methylbutanal oxime + 2 NADP+ + CO2 + 3 H2O | C00407 + 2 C00007 + 2 C00005 + 2 C00080 <=> C19491 + 2 C00006 + C00011 + 3 C00001 | C00407 | C19491 |
| R09404 | Aflatoxin B1 + Oxygen + NADPH + H+ <=> Aflatoxin Q1 + H2O + NADP+ | C06800 + C00007 + C00005 + C00080 <=> C19585 + C00001 + C00006 | C06800 | C19585 |
| R09405 | Aflatoxin B1 + Oxygen + NADPH + H+ <=> Aflatoxin M1 + H2O + NADP+ | C06800 + C00007 + C00005 + C00080 <=> C16756 + C00001 + C00006 | C06800 | C16756 |
| R09406 | Aflatoxin M1 + Oxygen + NADPH + H+ <=> Aflatoxin-M1-8,9-epoxide + H2O + NADP+ | C16756 + C00007 + C00005 + C00080 <=> C19594 + C00001 + C00006 | C16756 | C19594 |
| R09407 | Aflatoxin B1 + Oxygen + NADPH + H+ <=> Aflatoxin B1-endo-8,9-epoxide + H2O + NADP+ | C06800 + C00007 + C00005 + C00080 <=> C19595 + C00001 + C00006 | C06800 | C19595 |
| R09408 | Aflatoxin B1 + Oxygen + NADPH + H+ <=> Aflatoxin B1-exo-8,9-epoxide + H2O + NADP+ | C06800 + C00007 + C00005 + C00080 <=> C19586 + C00001 + C00006 | C06800 | C19586 |
| R09416 | 7,12-Dimethylbenz[a]anthracene + Oxygen + NADPH + H+ <=> 1a,11b-Dihydro-4,9-dimethylbenz[a]anthra[3,4-b]oxirene + H2O + NADP+ | C19488 + C00007 + C00005 + C00080 <=> C19489 + C00001 + C00006 | C19488 | C19489 |
| R09418 | trans-3,4-Dihydro-3,4-dihydroxy-7,12-dimethylbenz[a]anthracene + Oxygen + NADPH + H+ <=> (1aalpha,2beta,3alpha,11calpha)-1a,2,3,11c-Tetrahydro-6,11-dimethylbenzo[6,7]phenanthro[3,4-b]oxirene-2,3-diol + H2O + NADP+ | C19490 + C00007 + C00005 + C00080 <=> C19559 + C00001 + C00006 | C19490 | C19559 |
| R09421 | 4-(N-Nitrosomethylamino)-1-(3-pyridyl)-1-butanone + Oxygen + NADPH + H+ <=> 4-[(Hydroxymethyl)nitrosoamino]-1-(3-pyridinyl)-1-butanone + H2O + NADP+ | C16453 + C00007 + C00005 + C00080 <=> C19563 + C00001 + C00006 | C16453 | C19563 |
| R09423 | 4-(N-Nitrosomethylamino)-1-(3-pyridyl)-1-butanone + Oxygen + NADPH + H+ <=> 4-Hydroxy-4-(methylnitrosoamino)-1-(3-pyridinyl)-1-butanone + H2O + NADP+ | C16453 + C00007 + C00005 + C00080 <=> C19566 + C00001 + C00006 | C16453 | C19566 |
| R09424 | 4-(Methylnitrosamino)-1-(3-pyridyl)-1-butanol + Oxygen + NADPH + H+ <=> 1-(Methylnitrosoamino)-4-(3-pyridinyl)-1,4-butanediol + H2O + NADP+ | C19574 + C00007 + C00005 + C00080 <=> C19577 + C00001 + C00006 | C19574 | C19577 |
| R09425 | 4-(Methylnitrosamino)-1-(3-pyridyl)-1-butanol + Oxygen + NADPH + H+ <=> alpha-[3-[(Hydroxymethyl)nitrosoamino]propyl]-3-pyridinemethanol + H2O + NADP+ | C19574 + C00007 + C00005 + C00080 <=> C19580 + C00001 + C00006 | C19574 | C19580 |
| R09441 | 7,12-Dimethylbenz[a]anthracene + Oxygen + NADPH + H+ <=> 7-Hydroxymethyl-12-methylbenz[a]anthracene + H2O + NADP+ | C19488 + C00007 + C00005 + C00080 <=> C19561 + C00001 + C00006 | C19488 | C19561 |
| R09442 | 7,12-Dimethylbenz[a]anthracene + Oxygen + NADPH + H+ <=> 7,12-Dimethylbenz[a]anthracene 5,6-oxide + H2O + NADP+ | C19488 + C00007 + C00005 + C00080 <=> C19604 + C00001 + C00006 | C19488 | C19604 |
| R09451 | Hexadecanoic acid + NADPH + Oxygen + H+ <=> 16-Hydroxypalmitate + NADP+ + H2O | C00249 + C00005 + C00007 + C00080 <=> C18218 + C00006 + C00001 | C00249 | C18218 |
| R09452 | (9Z)-Octadecenoic acid + NADPH + Oxygen + H+ <=> 18-Hydroxyoleate + NADP+ + H2O | C00712 + C00005 + C00007 + C00080 <=> C19616 + C00006 + C00001 | C00712 | C19616 |
| R09453 | cis-9,10-Epoxystearic acid + NADPH + Oxygen + H+ <=> 9,10-Epoxy-18-hydroxystearate + NADP+ + H2O | C19418 + C00005 + C00007 + C00080 <=> C19620 + C00006 + C00001 | C19418 | C19620 |
| R09454 | Docosanoic acid + NADPH + Oxygen + H+ <=> 22-Hydroxydocosanoate + NADP+ + H2O | C08281 + C00005 + C00007 + C00080 <=> C19623 + C00006 + C00001 | C08281 | C19623 |
| R09460 | 16-Hydroxypalmitate + NADPH + Oxygen + H+ <=> 10,16-Dihydroxyhexadecanoic acid + NADP+ + H2O | C18218 + C00005 + C00007 + C00080 <=> C08285 + C00006 + C00001 | C18218 | C08285 |
| R09461 | 9,10-Dihydroxystearate + NADPH + Oxygen + H+ <=> 9,10,18-Trihydroxystearate + NADP+ + H2O | C19622 + C00005 + C00007 + C00080 <=> C19621 + C00006 + C00001 | C19622 | C19621 |
| R09492 | 2 Menaquinol + Oxygen <=> 2 Menaquinone + 2 H2O | 2 C05819 + C00007 <=> 2 C00828 + 2 C00001 | C05819 | C00828 |
| R09495 | N6-(L-1,3-Dicarboxypropyl)-L-lysine + H2O + Oxygen <=> L-2-Aminoadipate 6-semialdehyde + L-Glutamate + Hydrogen peroxide | C00449 + C00001 + C00007 <=> C04076 + C00025 + C00027 | C00449 | C04076 |
| R09504 | 2 Ubiquinol + Oxygen <=> 2 Ubiquinone + 2 H2O | 2 C00390 + C00007 <=> 2 C00399 + 2 C00001 | C00390 | C00399 |
| R09510 | Protein N6,N6-dimethyl-L-lysine + 2-Oxoglutarate + Oxygen <=> Protein N6-methyl-L-lysine + Succinate + Formaldehyde + CO2 | C05545 + C00026 + C00007 <=> C05544 + C00042 + C00067 + C00011 | C05545 | C05544 |
| R09511 | Protein N6-methyl-L-lysine + 2-Oxoglutarate + Oxygen <=> Protein lysine + Succinate + Formaldehyde + CO2 | C05544 + C00026 + C00007 <=> C02188 + C00042 + C00067 + C00011 | C05544 | C02188 |
| R09512 | Carbazole + NADPH + H+ + Oxygen <=> 2'-Aminobiphenyl-2,3-diol + NADP+ | C08060 + C00005 + C00080 + C00007 <=> C08061 + C00006 | C08060 | C08061 |
| R09513 | Methanesulfonic acid + NADH + H+ + Oxygen <=> Formaldehyde + NAD+ + Sulfite + H2O | C11145 + C00004 + C00080 + C00007 <=> C00067 + C00003 + C00094 + C00001 | C11145 | C00067 |
| R09514 | Urate + NADH + H+ + Oxygen <=> 5-Hydroxyisourate + NAD+ + H2O | C00366 + C00004 + C00080 + C00007 <=> C11821 + C00003 + C00001 | C00366 | C11821 |
| R09515 | Calcitriol + NADPH + H+ + Oxygen <=> Calcitetrol + NADP+ + H2O | C01673 + C00005 + C00080 + C00007 <=> C18231 + C00006 + C00001 | C01673 | C18231 |
| R09516 | Calcidiol + NADPH + H+ + Oxygen <=> Secalciferol + NADP+ + H2O | C01561 + C00005 + C00080 + C00007 <=> C07712 + C00006 + C00001 | C01561 | C07712 |
| R09517 | Anthranilate + FADH2 + Oxygen <=> 3-Hydroxyanthranilate + FAD + H2O | C00108 + C01352 + C00007 <=> C00632 + C00016 + C00001 | C00108 | C00632 |
| R09518 | Methane + Hydroquinone + Oxygen <=> Methanol + Quinone + H2O | C01438 + C15603 + C00007 <=> C00132 + C15602 + C00001 | C01438 | C00132 |
| R09519 | NH3 + Reduced acceptor + Oxygen <=> Hydroxylamine + Acceptor + H2O | C00014 + C00030 + C00007 <=> C00192 + C00028 + C00001 | C00014 | C00192 |
| R09549 | (+)-Germacrene A + NADPH + H+ + Oxygen <=> Germacra-1(10),4,11(13)-trien-12-ol + NADP+ + H2O | C16141 + C00005 + C00080 + C00007 <=> C19676 + C00006 + C00001 | C16141 | C19676 |
| R09555 | S-Benzoate coenzyme A + NADPH + H+ + Oxygen <=> 2,3-Dihydro-2,3-dihydroxybenzoyl-CoA + NADP+ | C00512 + C00005 + C00080 + C00007 <=> C19684 + C00006 | C00512 | C19684 |
| R09560 | 7-Chloro-L-tryptophan + Oxygen <=> 2-Imino-3-(7-chloroindol-3-yl)propanoate + Hydrogen peroxide | C19687 + C00007 <=> C19688 + C00027 | C19687 | C19688 |
| R09562 | Farnesylcysteine + Oxygen + H2O <=> 2-trans,6-trans-Farnesal + L-Cysteine + Hydrogen peroxide | C19691 + C00007 + C00001 <=> C03461 + C00097 + C00027 | C19691 | C03461 |
| R09565 | Gallate + Oxygen <=> (1E)-4-Oxobut-1-ene-1,2,4-tricarboxylate | C01424 + C00007 <=> C04434 | C01424 | C04434 |
| R09566 | Solavetivol + NADPH + H+ + Oxygen <=> Solavetivone + NADP+ + 2 H2O | C19711 + C00005 + C00080 + C00007 <=> C09737 + C00006 + 2 C00001 | C19711 | C09737 |
| R09567 | 3,4-Bis(7-chloroindol-3-yl)pyrrole-2,5-dicarboxylate + 4 Oxygen + 4 NADH + 4 H+ <=> Dichloroarcyriaflavin A + 2 CO2 + 6 H2O + 4 NAD+ | C19698 + 4 C00007 + 4 C00004 + 4 C00080 <=> C19699 + 2 C00011 + 6 C00001 + 4 C00003 | C19698 | C19699 |
| R09570 | L-Tryptophan + FADH2 + Cl- + Oxygen + H+ <=> 7-Chloro-L-tryptophan + FAD + 2 H2O | C00078 + C01352 + C00698 + C00007 + C00080 <=> C19687 + C00016 + 2 C00001 | C00078 | C19687 |
| R09571 | Dinoflagellate luciferin + Oxygen <=> Oxidized dinoflagellate luciferin + H2O + hn | C19704 + C00007 <=> C19705 + C00001 + C00205 | C19704 | C19705 |
| R09572 | L-Proline + 2-Oxoglutarate + Oxygen <=> cis-3-Hydroxy-L-proline + Succinate + CO2 | C00148 + C00026 + C00007 <=> C19706 + C00042 + C00011 | C00148 | C19706 |
| R09573 | 5-Epiaristolochene + 2 NADPH + 2 H+ + 2 Oxygen <=> Capsidiol + 2 NADP+ + 2 H2O | C19708 + 2 C00005 + 2 C00080 + 2 C00007 <=> C09627 + 2 C00006 + 2 C00001 | C19708 | C09627 |
| R09575 | Germacrene A acid + NADPH + H+ + Oxygen <=> Costunolide + NADP+ + 2 H2O | C19678 + C00005 + C00080 + C00007 <=> C09382 + C00006 + 2 C00001 | C19678 | C09382 |
| R09576 | Vetispiradiene + NADPH + H+ + Oxygen <=> Solavetivol + NADP+ + H2O | C12142 + C00005 + C00080 + C00007 <=> C19711 + C00006 + C00001 | C12142 | C19711 |
| R09577 | Solavetivol + NADPH + H+ + Oxygen <=> Solavetivone + NADP+ + 2 H2O | C19711 + C00005 + C00080 + C00007 <=> C09737 + C00006 + 2 C00001 | C19711 | C09737 |
| R09578 | L-Phenylalanine + 2 Oxygen + 2 NADPH + 2 H+ <=> (E)-Phenylacetaldoxime + 2 NADP+ + CO2 + 3 H2O | C00079 + 2 C00007 + 2 C00005 + 2 C00080 <=> C19714 + 2 C00006 + C00011 + 3 C00001 | C00079 | C19714 |
| R09579 | L-Phenylalanine + Oxygen + NADPH + H+ <=> N-Hydroxy-L-phenylalanine + NADP+ + H2O | C00079 + C00007 + C00005 + C00080 <=> C19712 + C00006 + C00001 | C00079 | C19712 |
| R09580 | N-Hydroxy-L-phenylalanine + Oxygen + NADPH + H+ <=> N,N-Dihydroxy-L-phenylalanine + NADP+ + H2O | C19712 + C00007 + C00005 + C00080 <=> C19715 + C00006 + C00001 | C19712 | C19715 |
| R09583 | L-Tryptophan + Oxygen + NADPH + H+ <=> N-Hydroxy-L-tryptophan + NADP+ + H2O | C00078 + C00007 + C00005 + C00080 <=> C19716 + C00006 + C00001 | C00078 | C19716 |
| R09584 | N-Hydroxy-L-tryptophan + Oxygen + NADPH + H+ <=> N,N-Dihydroxy-L-tryptophan + NADP+ + H2O | C19716 + C00007 + C00005 + C00080 <=> C19717 + C00006 + C00001 | C19716 | C19717 |
| R09586 | Pyrrole-2-carboxylate + NADH + H+ + Oxygen <=> 5-Hydroxypyrrole-2-carboxylate + NAD+ + H2O | C05942 + C00004 + C00080 + C00007 <=> C19721 + C00003 + C00001 | C05942 | C19721 |
| R09587 | Progesterone + Reduced ferredoxin + Oxygen <=> 15beta-Hydroxyprogesterone + Oxidized ferredoxin + H2O | C00410 + C00138 + C00007 <=> C19726 + C00139 + C00001 | C00410 | C19726 |
| R09601 | all-trans-8'-Apo-beta-carotenal + Oxygen <=> Retinal + (2E,4E,6E)-2,6-Dimethylocta-2,4,6-trienedial | C19728 + C00007 <=> C00376 + C19729 | C19728 | C19729 |
| R09602 | Pratensein + NADPH + H+ + Oxygen <=> 5-Hydroxypseudobaptigenin + NADP+ + 2 H2O | C10520 + C00005 + C00080 + C00007 <=> C19727 + C00006 + 2 C00001 | C10520 | C19727 |
| R09603 | Zeaxanthin + 2 Oxygen <=> Crocetin dialdehyde + 2 (3S)-3-Hydroxycyclocitral | C06098 + 2 C00007 <=> C19730 + 2 C19731 | C06098 | C19730 |
| R09604 | beta-Amyrin + Reduced acceptor + Oxygen <=> 24-Hydroxy-beta-amyrin + Acceptor + H2O | C08616 + C00030 + C00007 <=> C19732 + C00028 + C00001 | C08616 | C19732 |
|  | Sophoradiol + Reduced acceptor + Oxygen <=> Soyasapogenol B + Acceptor + H2O | C19733 + C00030 + C00007 <=> C08980 + C00028 + C00001 | C19733 | C08980 |
| R09671 | 4,4'-Diapolycopene + 4 Reduced acceptor + 4 Oxygen <=> 4,4'-Diapolycopenedial + 4 Acceptor + 6 H2O | C19797 + 4 C00030 + 4 C00007 <=> C19798 + 4 C00028 + 6 C00001 | C19797 | C19798 |
| R09682 | 9-cis-Epoxycarotenoid + Oxygen <=> Xanthoxin + 12'-apo-Carotenal | C19811 + C00007 <=> C13453 + C19812 | C19811 | C19812 |
| R09727 | 4,4'-Diaponeurosporene + 2 Reduced acceptor + 2 Oxygen <=> 4,4'-Diaponeurosporen-4-al + 2 Acceptor + 3 H2O | C16145 + 2 C00030 + 2 C00007 <=> C19839 + 2 C00028 + 3 C00001 | C16145 | C19839 |
| R09728 | 4,4'-Diaponeurosporen-4-al + Reduced acceptor + Oxygen <=> 4,4'-Diaponeurosporenic acid + Acceptor + H2O | C19839 + C00030 + C00007 <=> C16146 + C00028 + C00001 | C19839 | C16146 |
| R09747 | beta-Carotene + 2 NADH + 2 H+ + 2 Oxygen <=> Zeaxanthin + 2 NAD+ + 2 H2O | C02094 + 2 C00004 + 2 C00080 + 2 C00007 <=> C06098 + 2 C00003 + 2 C00001 | C02094 | C06098 |
| R09782 | Torulene + Oxygen <=> 4'-Apo-beta,psi-caroten-4'-al + 3-Methyl-2-butenal | C08613 + C00007 <=> C19892 + C07330 | C08613 | C19892 |
| R09784 | 2-Oxoglutarate + Oxygen <=> Ethylene + 3 CO2 + H2O | C00026 + C00007 <=> C06547 + 3 C00011 + C00001 | C00026 | C06547 |
| R09785 | 2-Oxoglutarate + L-Arginine + Oxygen <=> Succinate + CO2 + Guanidine + (S)-1-Pyrroline-5-carboxylate + H2O | C00026 + C00062 + C00007 <=> C00042 + C00011 + C17349 + C03912 + C00001 | C00062 | C03912 |
| R09786 | Dimethyl sulfide + Oxygen + NADH + H+ <=> Methanethiol + Formaldehyde + NAD+ + H2O | C00580 + C00007 + C00004 + C00080 <=> C00409 + C00067 + C00003 + C00001 | C00580 | C00409 |
