# Supplementary material for: The Impact of Oxygen on Metabolic Evolution: A Chemoinformatic Investigation
Source: PLoS Comput Biol. 2012 Mar 15;8(3):e1002426. doi: 10.1371/journal.pcbi.1002426 (PMC3305344; doi:10.1371/journal.pcbi.1002426)
Supplement: Table S5 — Property comparison between early and late anaerobic metabolites. (DOC) [file pcbi.1002426.s007.doc]

### Table S5 Property comparison between early and late anaerobic metabolites.

| **Descriptors** | **Mean values** | | ***P* value^d^** |
| --- | --- | --- | --- |
|  | Early anaerobic reactants  (n = 236) | Late anaerobic reactants  (n = 938) |  |
| MW^a^ | 373.72 | 294.87 | 6.15E-05 |
| AREA^a^ | 610.56 | 528.77 | 1.65E-03 |
| VOL^a^ | 937.80 | 792.87 | 1.44E-03 |
| AtomCount^a^ | 42.14 | 35.06 | 2.13E-03 |
| Carbon^b^ | 11.93 | 10.69 | 0.04 |
| Oxygen^b^ | 9.18 | 6.75 | 7.63E-08 |
| Nitrogen^b^ | 2.31 | 1.51 | 7.69E-06 |
| Sulfur^b^ | 0.14 | 0.09 | 0.04 |
| Phosphorus^b^ | 0.93 | 0.62 | 1.79E-04 |
| AlogP98^c^ | -1.79 | -0.55 | 3.53E-14 |
| PSA^a^ | 347.65 | 263.13 | 4.72E-11 |
| PV^a^ | 321.73 | 228.66 | 1.54E-09 |
| Acceptor^a^ | 10.00 | 7.07 | 5.19E-09 |
| Donor^a^ | 5.53 | 3.38 | 1.09E-11 |
| Hydrophobe^a^ | 1.52 | 1.41 | 0.46 |
| RingCount^a^ | 1.83 | 1.22 | 4.37E-08 |
| AromaticRings^b^ | 0.62 | 0.39 | 1.00E-03 |
| BondCount^a^ | 42.97 | 35.28 | 1.32E-03 |
| RotBonds^a^ | 11.08 | 8.44 | 5.13E-05 |
| Chiral^a^ | 4.25 | 2.72 | 3.36E-06 |

^a^ calculated with Sybyl 7.0

^b^ calculated with Pipeline Pilot

^c^ calculated with Cerius2

^d^ *t*-test
